# Using image segmentation models to analyse high-resolution earth observation data: new tools to monitor disease risks in changing environments

**DOI:** 10.1186/s12942-024-00371-w

**Published:** 2024-05-19

**Authors:** Fedra Trujillano, Gabriel Jimenez, Edgar Manrique, Najat F. Kahamba, Fredros Okumu, Nombre Apollinaire, Gabriel Carrasco-Escobar, Brian Barrett, Kimberly Fornace

**Affiliations:** 1 School of Biodiversity, One Health & Veterinary Medicine, University of Glasgow, Glasgow, Scotland UK; 2 School of Geographical & Earth Sciences, University of Glasgow, Glasgow, Scotland UK; 3 Sorbonne Université, Institute du Cerveau - ICM, CNRS, Inria, AP-HP, Paris, Inserm France; 4 Environmental Health and Ecological Sciences Department, Ifakara Health Institute, P. O. Box 53, Ifakara, Tanzania; 5 Centre National de Recherche et de Formation sur le Paludisme, Ouagadougou, Burkina Faso; 6 Health Innovation Laboratory, Institute of Tropical Medicine “Alexander von Humboldt”, Universidad Peruana Cayetano Heredia, Lima, Peru; 7 Saw Swee Hock School of Public Health, National University of Singapore and National University Health System, Singapore, Singapore

**Keywords:** Segment anything model, Mosquito-borne diseases, UAV, Drone, Remote sensing.

## Abstract

**Background:**

In the near future, the incidence of mosquito-borne diseases may expand to new sites due to changes in temperature and rainfall patterns caused by climate change. Therefore, there is a need to use recent technological advances to improve vector surveillance methodologies. Unoccupied Aerial Vehicles (UAVs), often called drones, have been used to collect high-resolution imagery to map detailed information on mosquito habitats and direct control measures to specific areas. Supervised classification approaches have been largely used to automatically detect vector habitats. However, manual data labelling for model training limits their use for rapid responses. Open-source foundation models such as the Meta AI Segment Anything Model (SAM) can facilitate the manual digitalization of high-resolution images. This pre-trained model can assist in extracting features of interest in a diverse range of images. Here, we evaluated the performance of SAM through the Samgeo package, a Python-based wrapper for geospatial data, as it has not been applied to analyse remote sensing images for epidemiological studies.

**Results:**

We tested the identification of two land cover classes of interest: water bodies and human settlements, using different UAV acquired imagery across five malaria-endemic areas in Africa, South America, and Southeast Asia. We employed manually placed point prompts and text prompts associated with specific classes of interest to guide the image segmentation and assessed the performance in the different geographic contexts. An average Dice coefficient value of 0.67 was obtained for buildings segmentation and 0.73 for water bodies using point prompts. Regarding the use of text prompts, the highest Dice coefficient value reached 0.72 for buildings and 0.70 for water bodies. Nevertheless, the performance was closely dependent on each object, landscape characteristics and selected words, resulting in varying performance.

**Conclusions:**

Recent models such as SAM can potentially assist manual digitalization of imagery by vector control programs, quickly identifying key features when surveying an area of interest. However, accurate segmentation still requires user-provided manual prompts and corrections to obtain precise segmentation. Further evaluations are necessary, especially for applications in rural areas.

**Supplementary Information:**

The online version contains supplementary material available at 10.1186/s12942-024-00371-w.

## Background

Global environmental change profoundly impacts the determinants of infectious disease transmission, with potentially devastating impacts on human health and well-being [1]. Changes in rainfall and global warming are associated with the emergence of vectors in new areas because of the increase of suitable habitats for their life cycle. Mosquito-borne diseases such as malaria, dengue, and Zika, are of particular interest because of their sensitivity to environmental variables [2–5]. Reports of rising disease cases have related them to different climate change events [6]. Some recent examples associated to climate disasters include malaria outbreaks in Mozambique and Pakistan caused by floods [7] and dengue outbreaks in Peru associated with heavy rains [8].

To reduce the burden of mosquito-borne diseases, control programs need to target interventions using environmental data to monitor changing risks. Over the last decades, there has been an increase in the use of remote sensing data to monitor ecological conditions, assess infectious disease risks, and identify priorities for control measures [9]. Typically, the data are gathered from diverse types of sensors carried by satellites, aircraft or small drones, which measure reflectance from the Earth’s surface [10]. The data can be analysed to identify landcover features of interest to monitor the composition of vector habitats, such as vegetation, water bodies, soil and infrastructure [11].

Historically, satellite data have been the most frequently used form of remote sensing data by disease control programs; these often include freely available satellite-based optical data from Landsat, Moderate Resolution Imaging Spectroradiometer (MODIS) and Sentinel-2 [12]. Moreover, processed products of these data have made remote sensing resources more accessible to the user by overcoming the challenges associated with managing complex satellite image processing steps and landcover classification. However, despite increasing availability and constantly improving spectral resolution, there are still substantial barriers to their use by disease control programs. Cloud coverage limits the availability of usable data over time especially during the rainy season [13]. This can be a major limitation to obtaining images in peak transmission periods as mosquito populations increase due to the presence of semi-permanent aquatic habitats in the rainy season [14]. Moreover, low spatial resolution restricts focused studies, such as the understanding of micro-habitats that need a finer scale spatial resolution only available in commercial satellites [15].

These limitations have led to a rise in targeted mapping using low-cost drones to collect high- resolution imagery at specific locations and times. Different from satellite acquisition, data collection using drones needs to ensure compliance with ethical considerations and obtaining consent from communities [16]. Many recent studies have conducted image acquisition using small low-cost drones to evaluate the feasibility of using this technology for larval source management, aiming to identify water bodies where malaria mosquito vectors breed [17–19]. However, in contrast to satellite-based information, drone image pre-processing and classification need to be performed by the user. The automation of identifying specific features over large image datasets can be challenging and requires access to specialized expertise, software, and computing infrastructure. These capabilities can be provided by technology solutions such as Zzapp for malaria control programmes [20]. However, many of these still rely largely on manual digitization which can be a time-consuming task.

Advances in computer vision have helped in the automatic identification of specific objects in high-resolution remote sensing images and the integration of these methods in GIS software allows end users to apply them without advanced programming skills. The application of computer vision methods for land class identification associated with mosquito habitats range from the classification of pixels in spectral bands (e.g. Carrasco et al. [17]), object-based recognition for land classification (Stanton et al. [18]) to segmentation using deep learning models focusing on land classes such as crops and houses (e.g. Trujillano et al. [21]). Despite the high accuracy using these techniques [22–24], most are based on supervised learning and their model performance depends on training with manually annotated datasets to learn the patterns of the class of interest. In the past few years, convolutional neural networks (CNNs) for segmentation have been widely used for land classification in remote sensing images [21, 25]. Recent architectures such as visual transformers are currently being preferred for improved segmentation performance, but these approaches require extensive training data, limiting their utility [26].

Foundation models, models trained on broad datasets, have significantly advanced this field and have shown impressive results [27]. The Segment Anything Model (SAM), a foundation model developed by Meta AI, is a generalizable trained model with the capacity to extract features from images independently of their domain [28]. This model can be run using the open-source Samgeo python package [29] which simplifies the way users enter text and spatial prompts to guide SAM segmentation for geospatial data. However, despite the utility of these foundation models, these tools have not been widely applied for infectious disease epidemiology.

Therefore, this study aims to assess the capability of new computer vision advances by measuring the segmentation performance of key land features that are relevant for disease surveillance in high-resolution drone images. For this purpose, we explored SAM, a trained segmentation model, using the Samgeo python package to evaluate if this ready-to-use tool can reduce the current barrier of using complex computer vision models by non-specialists. Moreover, we performed a comparative analysis using drone images previously acquired in three malaria-endemic areas: Africa, South America, and Southeast Asia to evaluate the performance in different geographic contexts and to identify the landscape characteristics in which this type of approach could be useful. The development of new user-friendly technologies can benefit non-expert image processing users in identifying key features in large datasets of images and reduce the amount of time and effort required for human annotation, leading to faster response times to prevent potential disease outbreaks.

## Data and methods

### Study sites

**Fig. 1:**
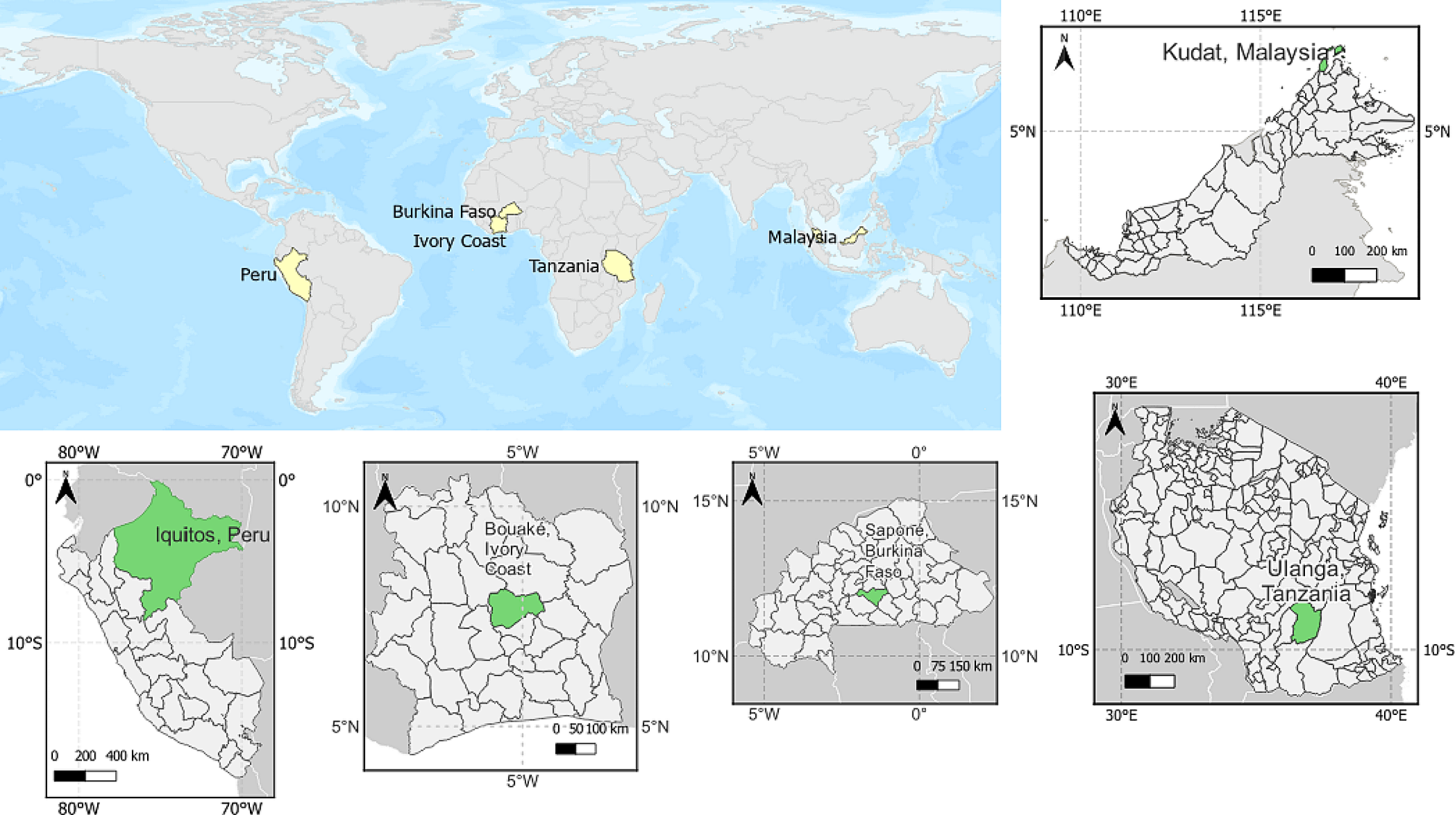
Geographic location of the study sites in Peru, Burkina Faso, Ivory Coast, Malaysia, and Tanzania

We performed a secondary data analysis using drone imagery collected from five different sites (see Fig. 1). Even though all these areas are malaria-endemic, the habitats and species vary from one location to another.

In South America, images were collected in the surrounding rural villages of Iquitos, Peru, in the Amazon region. These areas are recognized for high dengue and malaria transmission rates [30]. The primary mosquito vector in this region is *Nyssorhynchus darlingi*, using a variety of aquatic habitats from natural to human made water bodies. The peak transmission period falls between January to June aligning with the rainy season [17].

In sub-Saharan Africa, we obtained drone images from three malaria-endemic locations. This included areas around Bouaké, Ivory Coast, where peak malaria transmission occurs in the rainy season between May and October. The potential breeding sites are irrigated crops and large vegetated water bodies [31]. Similarly, Saponé, Burkina Faso has a seasonal malaria transmission with a rainy season that extends from June to October. The larval breeding sites are water reservoirs such as dams, canary pots, worn tyres and small water reservoirs. The Ulanga distric, in south-eastern Tanzania, experiences its rainy season from March to May. The dominant vector is *Anopheles funestus* and the breeding sites are river streams, ground pools and dug pits [32].

Lastly, in Southeast Asia, drone images were obtained from Northern Sabah, Malaysian Borneo where zoonotic malaria transmission is endemic. This is a tropical area with variable rainfall and the dominant vector is *Anopheles balabacensis, which* breeds in small water bodies under forest cover [33]. We used RGB drone imagery acquired by different platforms and settings across these rural sites as described in Table 1. Together, these sites represent a diverse group of landscapes with on-going malaria transmission.

### Key land classes of interest

We focused our study on the recognition of water bodies and human settlements given their relevance when monitoring potential aquatic habitats of mosquitoes and targeting control measures based on high-resolution Earth observation (EO) data. The preferred mosquito breeding sites depend on the species and the specific local context, ranging from man-made reservoirs to natural stagnant water bodies or slow-flowing river streams. We therefore evaluated the capability to detect different types and sizes of waterbodies.

Human settlements are also important in the spread of diseases carried by mosquitoes and should be considered when assessing population-level risk as well as individual-level risk from mosquito bites. Across the different study sites, the characteristics of housing are diverse, particularly in rural environments where variations in roof types and spatial separation were significant. The classes identified in each study site and the technical details and methods of the drone acquisitions are detailed in Table 1.

### Dataset

To perform a comparison across sites, we extracted 14 representative samples from each study area (reported in the Supplementary Information) considering all the collected orthomosaics. The samples sizes were 120 × 120 m and 60 × 60 m, and the selection was performed manually, targeting the areas where water bodies and human settlements were present. The patch size was selected to include the objects of interest; however, samples with no classes were also selected. Out of the 70 patches, 47 samples contained buildings and 44 contained water.

**Table 1:** Description of site characteristics and data acquisition parameters

Site	Characteristics		Image collection		
	Human-settlement	Water bodies	Equipment	Date range	Resolution (cm/pixel)
Iquitos, Peru	Clustered housing. Corrugated metal roofs.	Man-made fishponds. Large stagnant water bodies.	Mavic 2 Pro	Aug - Oct 2021	2–10
Bouaké, Ivory Coast [31]	Separated houses. Corrugated metal, and straw roofs.	Rains puddles in vegetated areas.	DJI Phantom 4 Pro	Nov 2018 – Nov 2019	2
Saponé, Burkina Faso [21]	Compounds with multiple houses. Thatched rounded huts. Corrugated metal roofs.	Vegetated and non-vegetated large water bodies. Turbid shallow puddles.	DJI Phantom 4 Pro / Sensefly eBee	Jun to Aug 2021	2–10
Ulanga, Tanzania	Isolated thatched houses.	Rain puddles. Turbid ground pools.	DJI Phantom 4 Pro	May 2023	6
Kudat, Malaysia [34]	Isolated houses. Mixed roof types: red, metal and concrete.	River streams. Fishponds. Reservoirs.	Sensefly eBee	2014–2019	10

### The Samgeo package

Recently, Visual Transformers (ViT) have outperformed CNN-based models in object detection and segmentation tasks [35]. The key difference lies in how they compute the features of an image. While CNNs use convolutional layers to extract local information about the images, ViT divides the image into smaller patches called tokens and learns the relationships between them, allowing it to capture both local and global information. However, this process is computationally expensive during training and requires larger datasets to achieve meaningful performance.

The Segment Anything Model (SAM) [28] assembles several blocks that perform specific tasks (e.g. segmentation and detection), and this model uses Vision Transformers to encode the input image into its embeddings, which is a numerical vector representation of an image. The model is capable of segmenting different areas or objects; however, other information such as spatial points and bounding boxes information can also be given to the model to indicate the object to be segmented. The final block comprises a decoder that maps the segmentation output mask to the original image size.

SAM’s segmentation capability can be integrated into large systems of different domain-specific applications if an object identification model is available. The Samgeo Python package simplifies the process of preparing geospatial data as input for the SAM model and generating georeferenced output masks in GIS-compatible formats. This package also allows text prompt for segmentation which is supported by the Grounding Dino object detector model [36]. This transformer-based detector processes an image and a text input to locate the specified object in the image within a bounding box. The Samgeo package then uses the bounding box as an input for the SAM model to perform the segmentation. We tested the capacity of this text-prompt feature to detect and segment landscape features.

### Segmentation guided by spatial point prompts

**Fig. 2:**
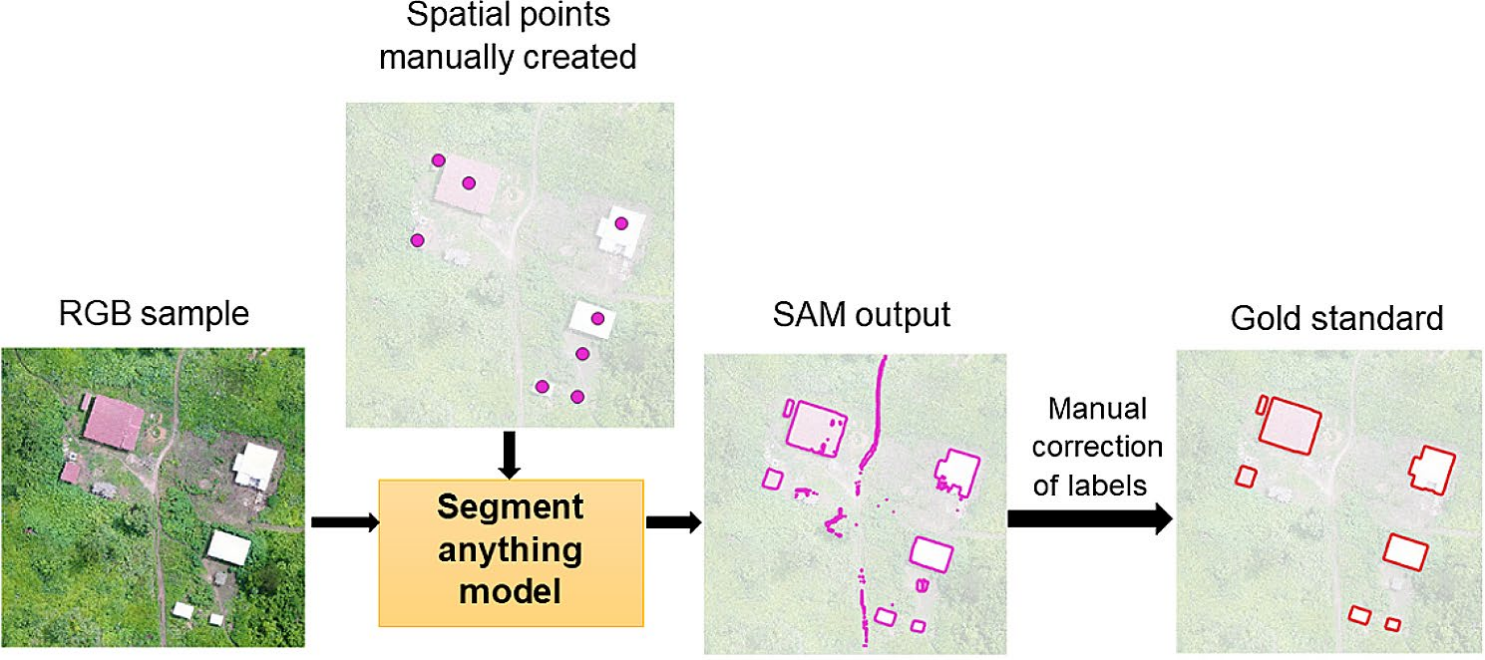
Workflow for gold standard generation

We defined the gold standard to represent the most accurate identification of the land class, performed by visually inspecting the acquired images. To create the gold standard, we used SAM’s guided object segmentation capability to assist this process (Fig. 2). First, we evaluated each patch to determine the presence of water bodies or buildings. Then we created a shapefile for each identified class using QGIS software, version 3.32 [37]. We created a point for each water body and, for buildings we created a point for every discernible roof. This approach was adopted since clear delineation of house boundaries was challenging in some sites. All the points were positioned approximately at the centre of the objects. The segmentation result was exported as a shapefile, and to obtain the gold standard, manual corrections were made to rectify errors such as boundaries inaccuracy, missed objects or false positives.

### Text prompt segmentation

For the text prompt experiments, we defined a list of text prompts for the class of interest. We tested a single word: “building”, “house”, “roof”, “puddles”, “ponds” and “water body”, and ensembled words: “house.thatch.dwelling.roof.building”, and “puddle.water body.pond.fishponds”. A GPT model, ChatGPT-4 [38] was used to generate prompts in the following 4 steps:

Step 1. Upload three images from sites and ask ChatGPT to describe them.
  Query prompt: “Describe the uploaded image”.

Step 2. Ask for a text prompt for water bodies and houses based on the images.
  Query prompt: “Generate a ‘text prompt’ for the SAM model in order to segment buildings in similar images to the one uploaded.”
  Query prompt: “Generate a ‘text prompt’ for the SAM model in order to segment water bodies in similar images to the one uploaded.”

Step 3. Ask to refine the given prompt to only include keywords.
  Query prompt: “Consider that the prompt for the model should be a list of keywords, each separated by a period. Generalize it to different types of buildings.”
  Query prompt: “Consider that the prompt for the model should be a list of keywords, each separated by a period. Generalize it to different types of water bodies.”

Step 4. Manually remove keywords that are not meaningful.

**Fig. 3:**
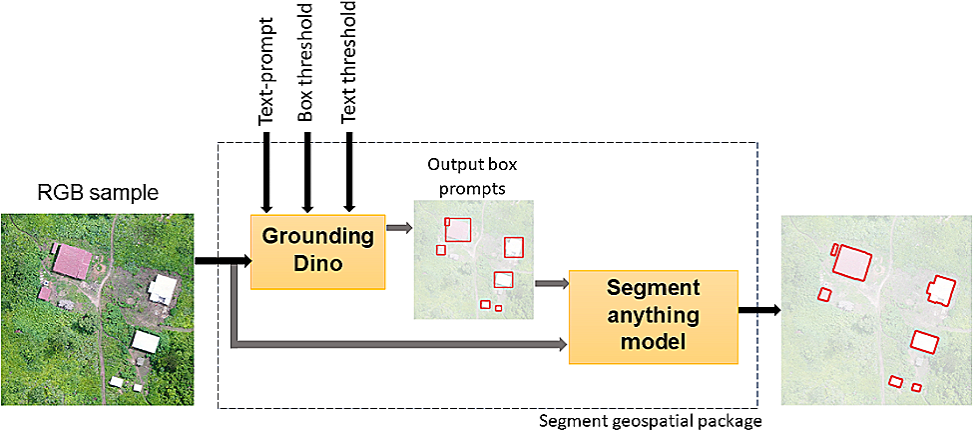
Text prompt segmentation pipeline

After filtering words such as aerial view, rural and shadow, we obtained for buildings the GPT prompts: “buildings.roofs.reflective surfaces.matte surfaces.geometric shapes.metallic.tiles.thatch” and for water “water.ponds.lakes.rivers.streams.murky water.clear water.reflections”. Details about the questions and answers obtained using ChatGPT are reported in the Supplementary Information. In addition to the text-prompt, two other inputs were needed for the Grounding Dino model, these were the box and text threshold that can be selected in the range from 0 to 1. By default, the model outputs 900 bounding boxes and the box threshold serves to filter the number of boxes as the threshold approaches to 1. Figure 3 shows the inputs and outputs of each model to perform the segmentation.

All the datasets were run using these combinations of prompts on a High-Performance Computing (HPC) system with 2x AMD 7543 Processors @2.8 Ghz, 256Gb of RAM and a Nvidia A40 (48 GB) GPU.

### Metrics used to measure performance

The segmentation and object detection performance were evaluated quantitatively using the Dice similarity coefficient for class segmentation. This metric is commonly used to assess segmentation performance in vision computer tasks [39]. The Dice coefficient measures the overlap between two regions A and B, where A represents the gold standard and B the predicted segmentation. Shapefiles were converted to binary raster masks for this computation. The coefficient was calculated as follows:

1 $$Dice\left(A,B\right)= \frac{2\mid \text{A}\cap \text{B}\mid }{\mid \text{A}\mid +\mid \text{B}\mid }$$

Where $\mid \text{A}\cap \text{B}\mid$ is the number of elements common to both A and B (i.e. the intersection of A and B), ∣A∣ is the number of elements in set A and ∣B∣ is the number of elements in B, and the Dice coefficient is measured in the range of 0–1. Additionally, to evaluate the robustness of the prediction we computed the bootstrap confidence interval (CI) [40] with 5000 resamples for each text prompt and box threshold.

**Fig. 4:**
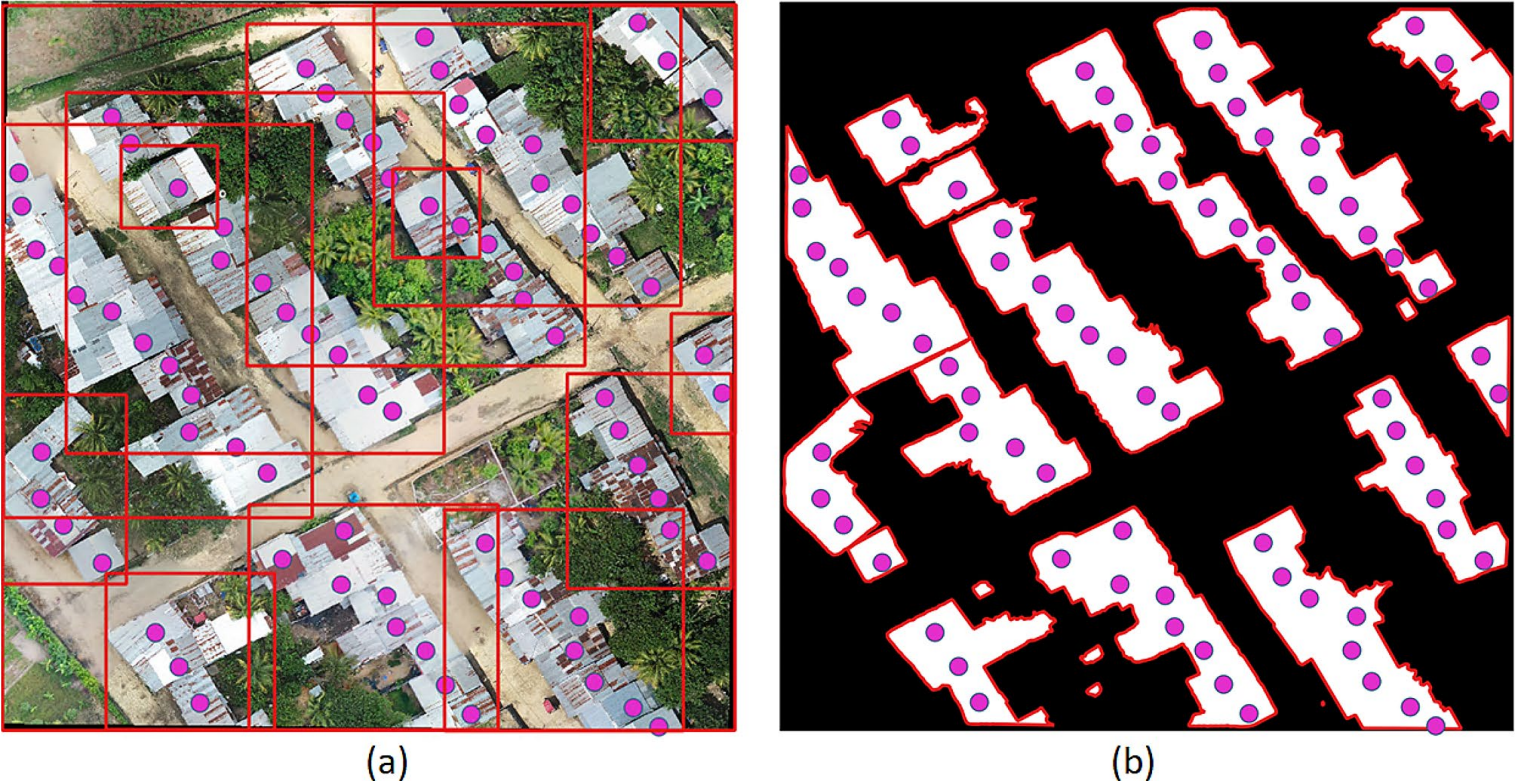
Iquitos, Peru. (**a**) Grounding Dino bounding boxes (**b**) SAM output segmentation

​For the object detection evaluation, we did not use metrics such as the Intersection over Union (IoU) because some sites contained a large number of objects causing the object bounding boxes to overlap as shown in Fig. 4(a). Instead, we used as a reference the points manually assigned to each object to validate whether they were identified by SAM’s predicted polygon output as shown in Fig. 4(b). We considered a true positive (TP) if the point was inside the model predicted polygon, a false positive (FP) if a polygon was predicted and no point was inside, and a false negative (FN) if a point did not match any polygon. The object accuracy detection was measured by the Positive predictive value (PPV) and True positive rate (TPR). Calculated by the following equations: PPV = TP/(TP + FP) and TPR = TP/(TP + FN).

## Results

### Spatial prompt segmentation

**Fig. 5:**
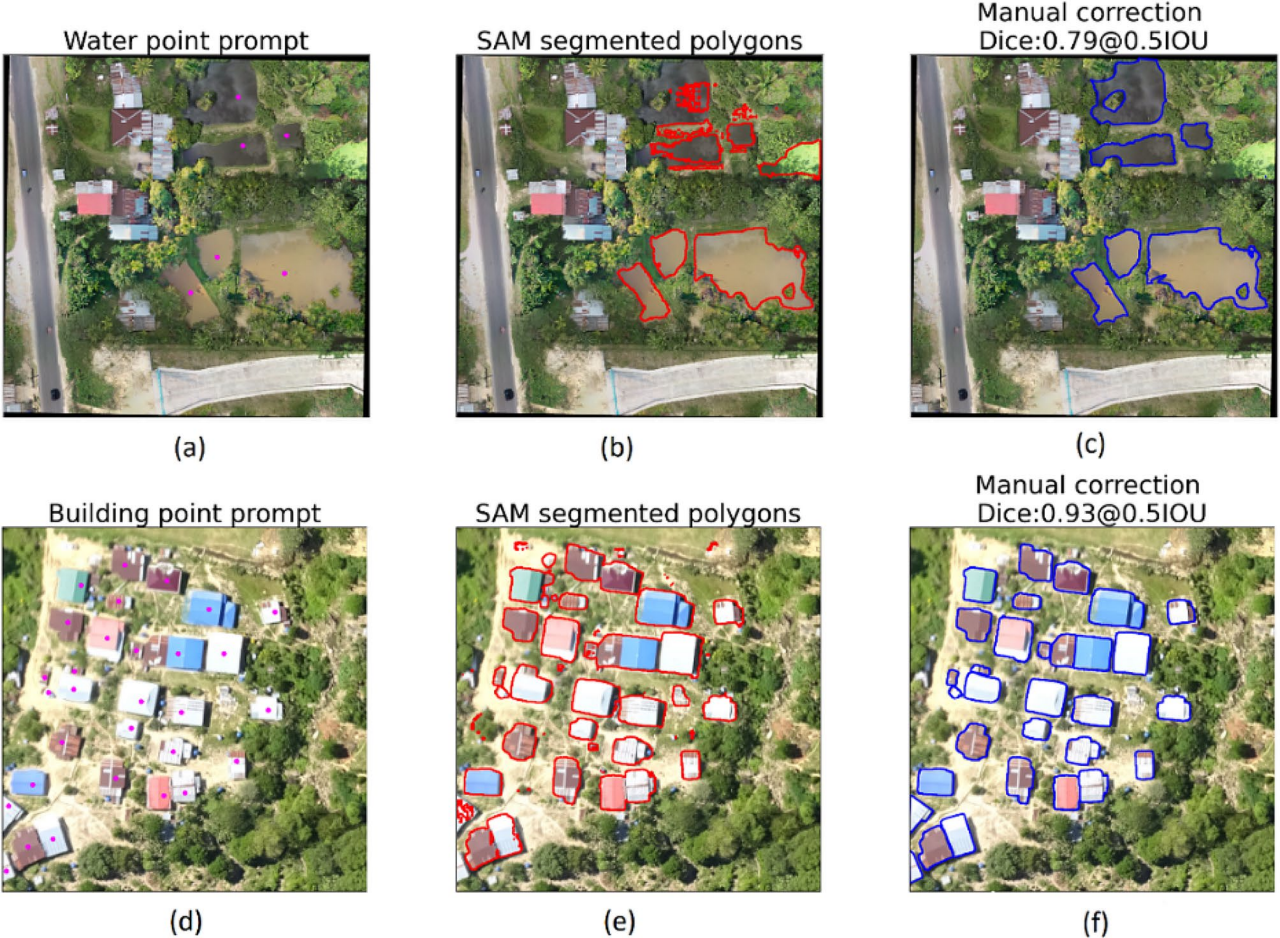
(**a**) Iquitos, Peru and (**d**) Malaysia show point prompts in magenta used for water and buildings segmentation. (**b**) and (**e**) show the output of SAM in red polygons. (**c**) and (**f**) show the gold standard in blue polygons

An average Dice coefficient value for all samples was calculated using point prompts obtaining 0.67 for buildings and 0.73 for water bodies. Figure 5 presents examples of SAM segmentation output in the study sites. In Fig. 5(b), water bodies at the top of the patch were not correctly segmented and manual correction was needed to rectify the shapes, which impacted the accuracy (Dice coefficient = 0.79). Conversely, Fig. 5(a) shows a sample where the majority of houses were accurately segmented. Small polygons around the houses were deleted and minor boundary corrections on the bottom houses were performed, reflecting in a higher Dice coefficient of 0.93.

**Fig. 6:**
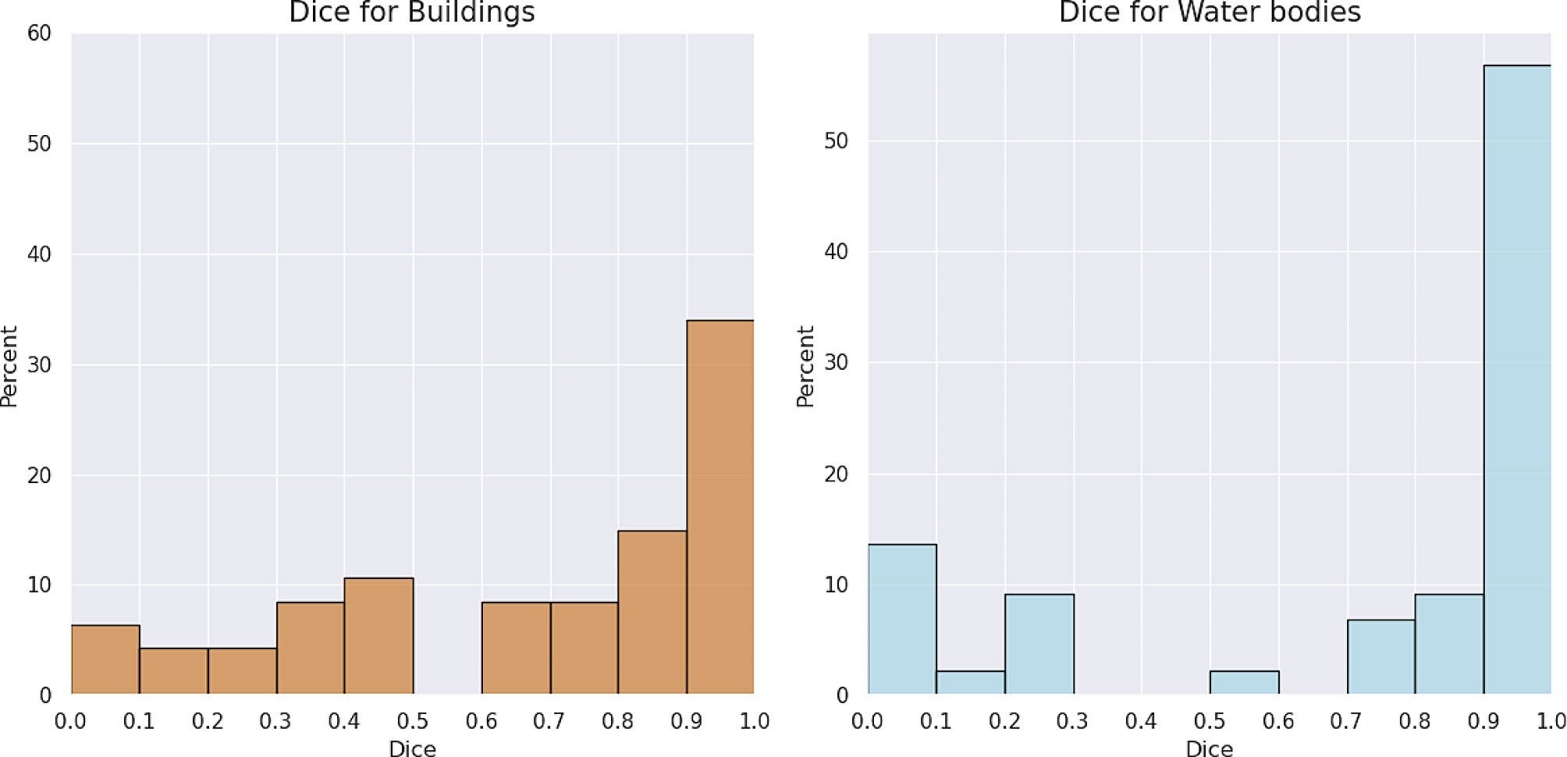
Dice coefficient histograms per class

**Fig. 7:**
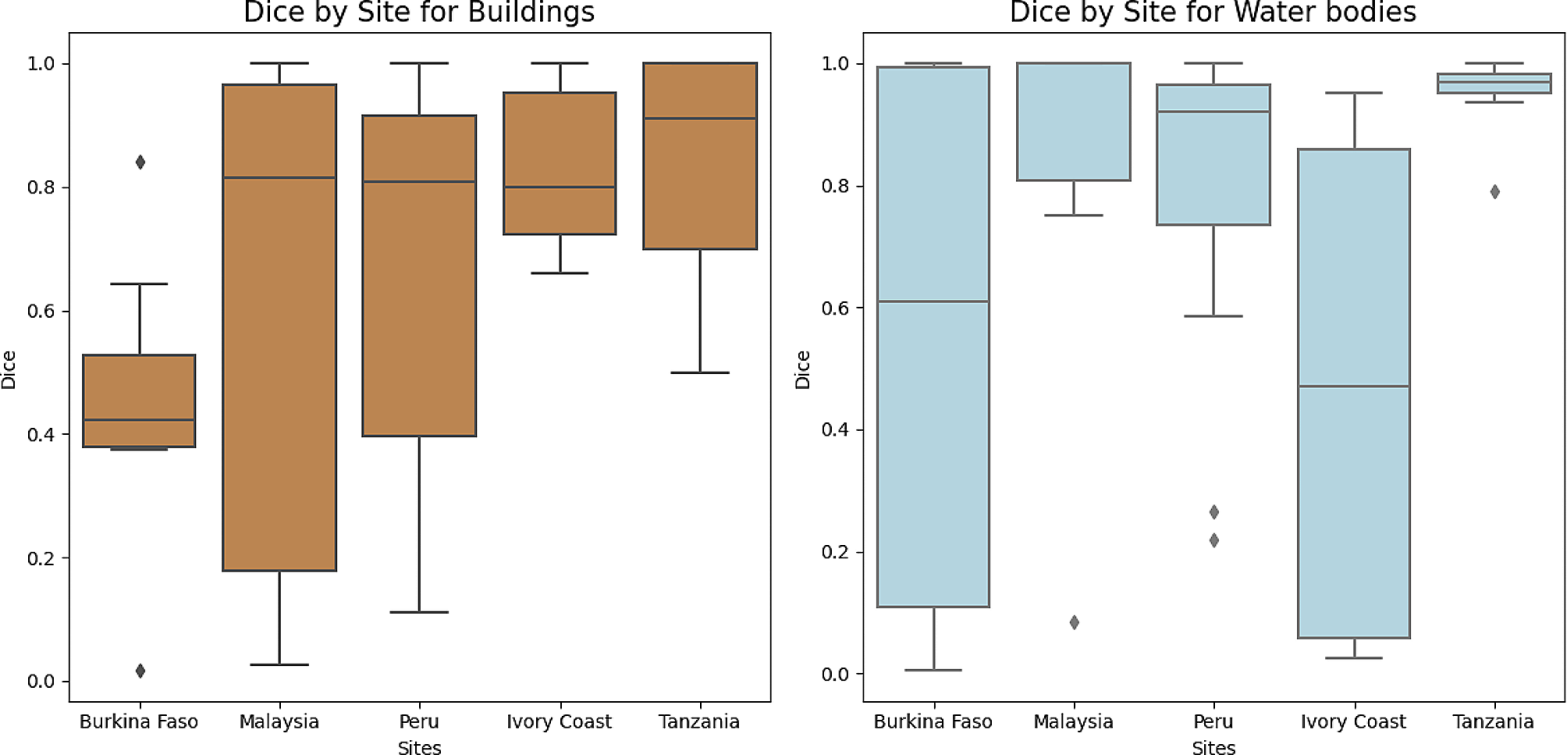
Boxplot of Dice coefficients for each class by site

In the case of buildings, 49% of the patches had a Dice coefficient value above 0.8 and for water bodies 65%, showing that minor corrections were needed in the SAM generated shapefiles (Fig. 6). On the other hand, 11% of the buildings and 16% of the water bodies needed to be almost fully manually digitalized. In general, there was a better performance of human settlements segmentation in the sites of Tanzania and Ivory Coast, and for water segmentation, Malaysia, Peru, and Tanzania were the sites where fewer corrections were required (Fig. 7). The accuracy of class boundary identification varied across the other sites.

### Text prompt segmentation

Text prompts showed variable performance depending on the class, prompts used, and location. Results from 0.3 to 0.4 box threshold are summarized in Table 2 while other values are reported in the Supplementary Information. Overall, the highest Dice coefficient for buildings was 0.725 with CI [0.724 0.725], achieved using the word “house” and for water bodies, it was 0.695 with CI [0.694 0.696] using the mix of different words; predictions were made using box thresholds of 0.4 and 0.3 respectively. Regarding object detection, the metrics in general show a low PPV performance due to a high number of small polygons generated as false positives. For the buildings class, the “house” text prompt with a 0.3 box threshold resulted in the highest TPR; nevertheless, for PPV, a 0.4 box threshold showed a marginal improvement. In the case of water bodies, the word “ponds” with a 0.3 threshold reported the higher value.

**Table 2:** Segmentation and object detection metrics according to text prompts

		Box threshold							
		**0.3**				**0.4**			
	Text prompt	CI	Dice std.	TPR	PPV	CI	Dice std.	TPR	PPV
Buildings	Building	0.603 [0.603 0.604]	0.420	0.518	0.446	0.620 [0.619 0.621]	0.409	0.318	0.482
	House	0.716 [0.715 0.716]	0.362	0.554	0.515	0.725 [0.724 0.725]	0.350	0.406	0.537
	Roof	0.379 [0.379 0.380]	0.415	0.360	0.448	0.462 [0.461 0.463]	0.445	0.176	0.320
	Ensembled	0.635 [0.634 0.636]	0.412	0.413	0.478	0.468 [0.467 0.469]	0.441	0.093	0.293
	GPT prompt	0.351 [0.350 0.351]	0.404	0.251	0.282	0.369 [0.368 0.370]	0.446	0.041	0.149
Water	Puddles	0.640 [0.639 0.640]	0.441	0.288	0.304	0.537 [0.536 0.538]	0.476	0.103	0.200
	Ponds	0.446 [0.445 0.446]	0.449	0.393	0.316	0.546 [0.545 0.547]	0.460	0.184	0.242
	Water body	0.375 [0.374 0.376]	0.440	0.371	0.310	0.522 [0.521 0.523]	0.463	0.227	0.249
	Ensembled	0.695 [0.694 0.696]	0.424	0.325	0.308	0.565 [0.565 0.566]	0.471	0.131	0.184
	GPT prompt	0.577 [0.576 0.578]	0.470	0.168	0.177	0.372 [0.371 0.373]	0.484	0.000	0.000

**Fig. 8:**
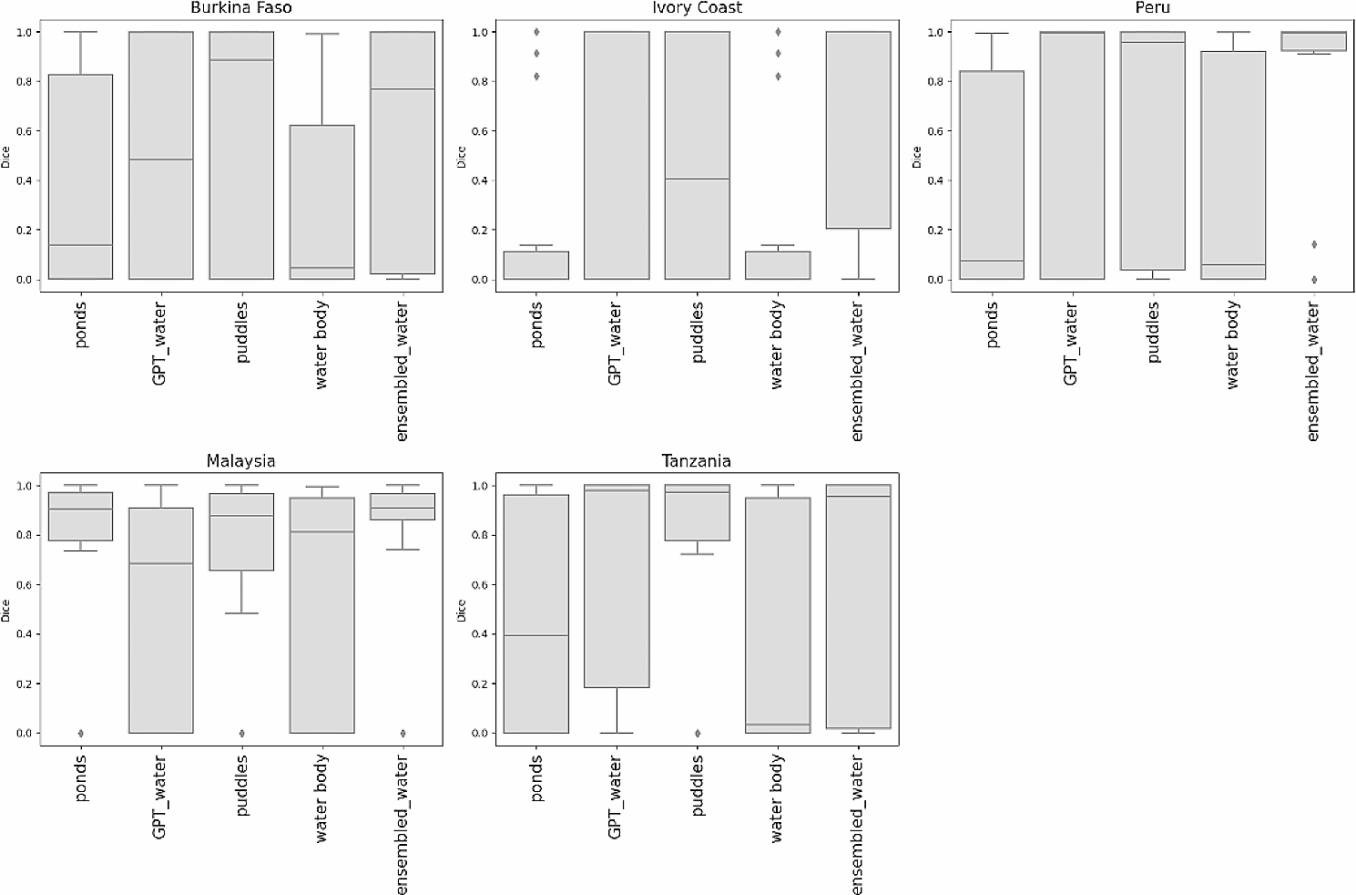
Dice coefficient for each site according to the water text prompts used with 0.3 box threshold

**Fig. 9:**
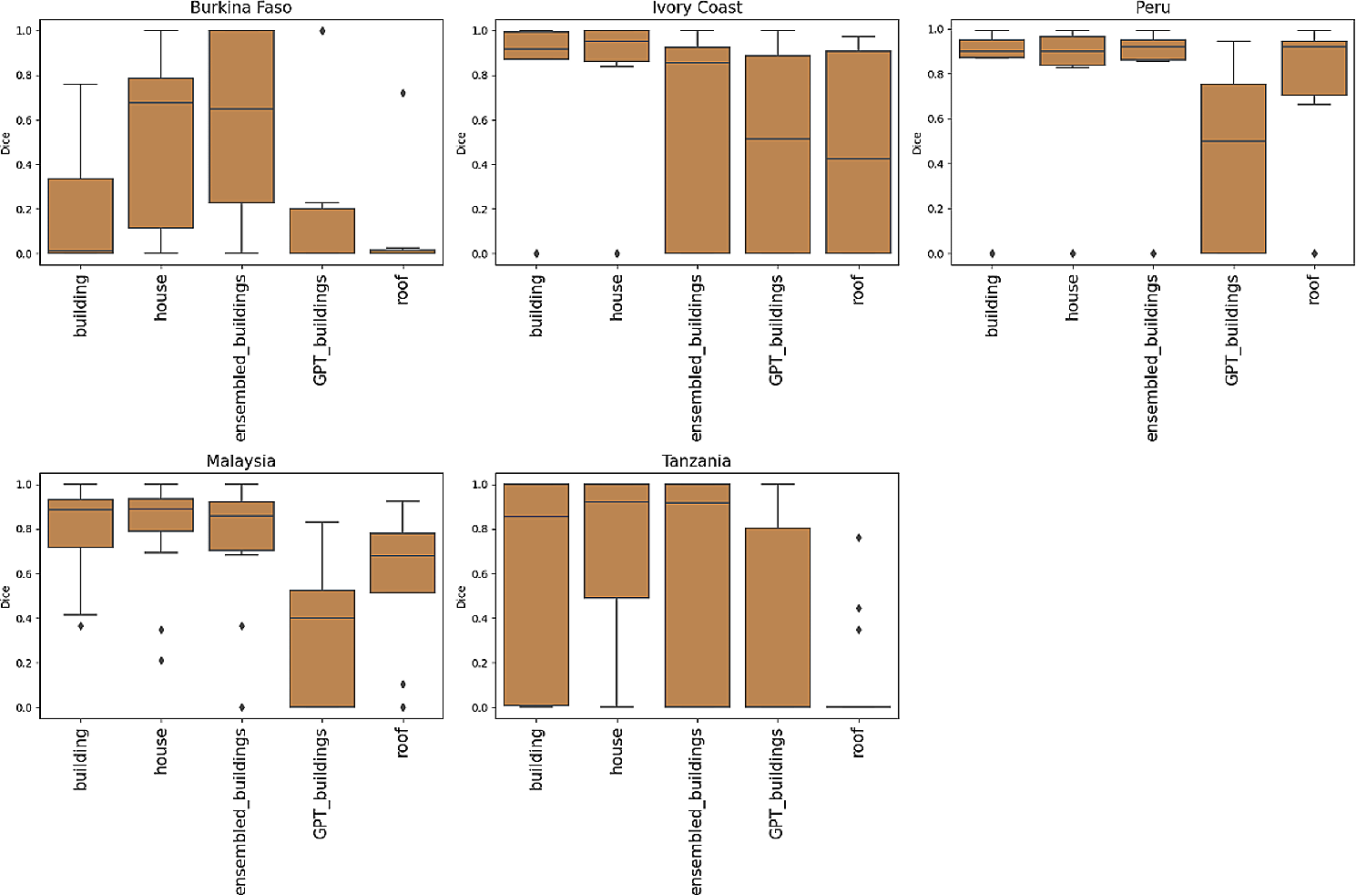
Dice coefficient for each site according to the building text prompts used with 0.3 box threshold

Figures 8 and 9 present boxplots of the Dice coefficient per site. The performance of the words selected for the text prompt segmentation was not consistent across all sites. Considering the water body class, an ensembled choice of words performed best in images from Peru and Malaysia, whereas for Tanzania, the word “puddles” was the most effective. The results from Burkina Faso showed a marked variability and those from Ivory Coast showed a low performance in all cases. For buildings, “house” had a higher average Dice coefficient for Ivory Coast and Malaysia, although in Peru an ensembled combination yielded the best performance. Building segmentation results from Tanzania were highly variable and all the text prompts used for images from Burkina Faso performed poorly.

**Fig. 10:**
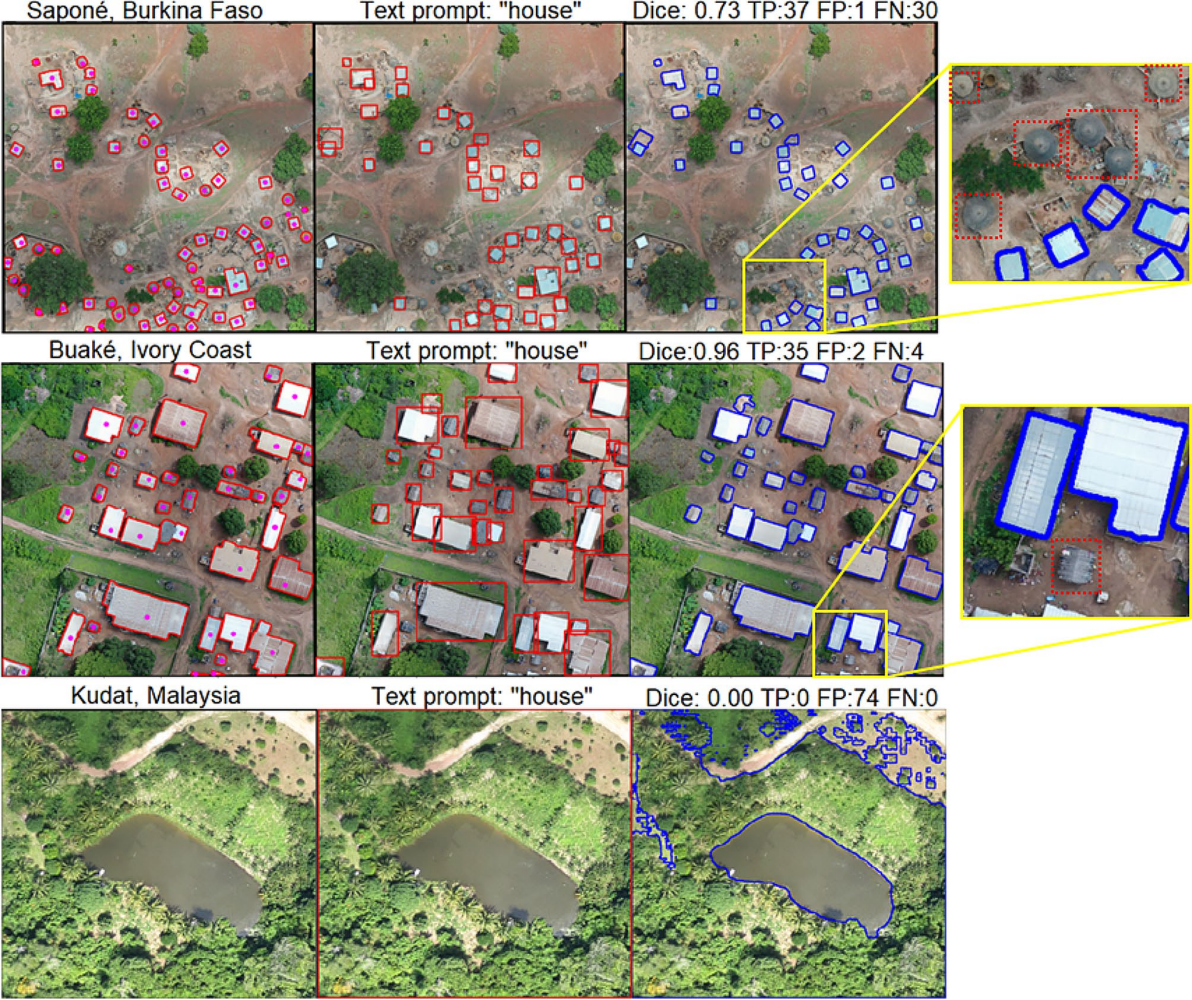
Results using a 0.3 Box threshold. The image on the left corresponds to the buildings gold standard, the middle image shows the Grounding Dino bounding boxes in red and the right figure shows in blue SAM’s output. The sample from Kudat does not contain houses

**Fig. 11:**
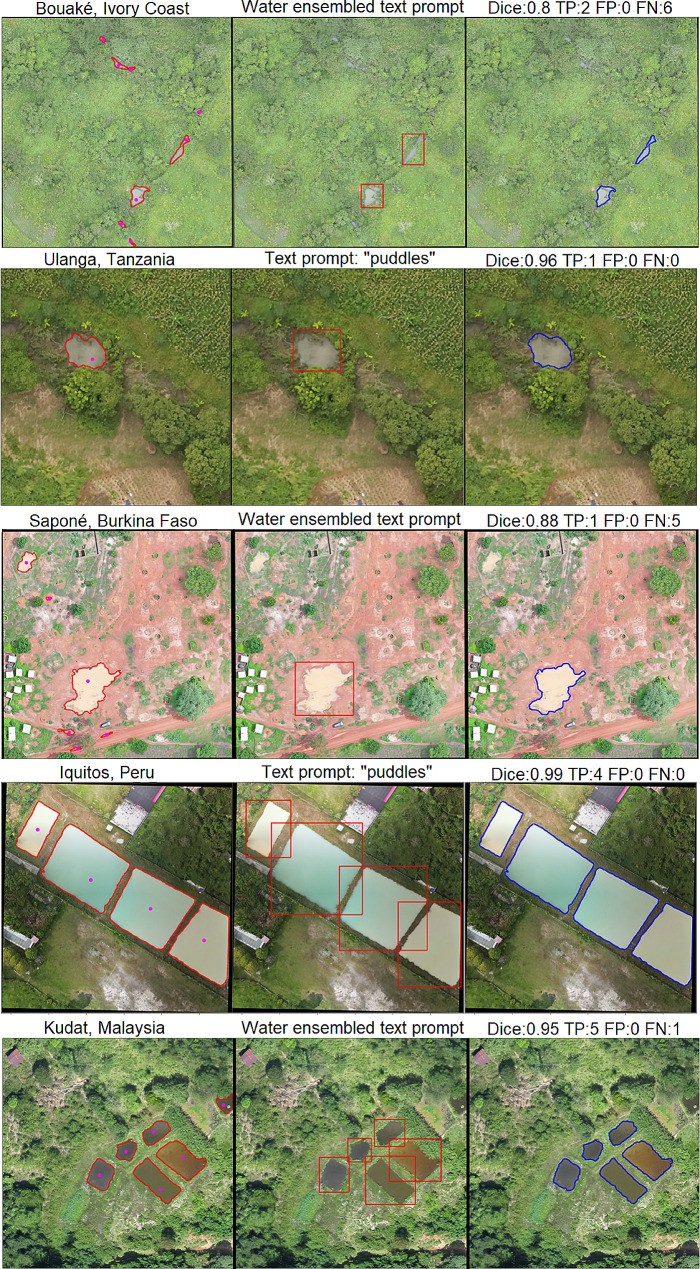
Results using a 0.3 Box threshold. The image on the left corresponds to the water body gold standard, the middle image shows the Grounding Dino bounding boxes in red and the right figure shows in blue SAM’s output

Figure 10 shows examples of houses that the Grounding Dino model missed. These included rural dwellings such as thatched rounded huts in Burkina Faso and straw roof structures in Ivory Coast. The example from Malaysia, without any house structure, shows the bounding box aligned with patch boundaries, leading SAM to segment the areas within. These large false positive polygons resulted in a low Dice coefficient, impacting the average value. Figure 11 shows the variability of colour, shape, and extension of water bodies which resulted in variable segmentation performance. Small puddles in vegetated areas and along roads in Burkina Faso and Ivory Coast were undetected, in contrast to larger areas with clear boundaries, such as fishponds in Peru or ground pools in Tanzania, which were identified despite of their varying colours. Additional results are reported in the Supplementary Information.

## Discussion

In this study, we explored cutting-edge techniques for segmentation and applied them to high-resolution drone imagery from five study sites across the globe to identify water bodies and human settlements that are relevant classes for vector-borne disease control. Foundation models, such as SAM, have shown potential to rapidly segment and identify key land classes from high-resolution imagery, enabling the targeting of disease control activities. We found that the segmentation accuracy was highly dependent on the specific features and landscape characteristics, leading to variable performance by methods and sites. While these models can be a valuable tool to support the analysis of high-resolution imagery, there is still a need for manual corrections and user input.

Despite the variable performance, the use of the Samgeo package can significantly reduce the effort required for manual annotation of drone imagery. Approximately 65% of the water bodies and 50% of buildings in tested patches required minimal correction (Dice coefficient > 0.8) following SAM prediction, using a single user point for segmentation. This approach using either a point or a word for segmentation is relatively straightforward compared to other supervised learning methods such as CNNs [21] and random forest [17, 18].

Guided segmentation, using points or text prompts, achieved varying performance based on class characteristics, such as the clear boundaries of the object and its extension within the patch. Therefore, well-delineated water bodies such as fishponds, rivers, and large water bodies were segmented more effectively, whereas the performance varied in samples containing small puddles on roads or in grass. Regarding the segmentation of houses, typical urban houses were segmented more accurately than rural buildings such as rounded huts. The identification accuracy was not consistent even within the same study site. According to Zhang [41], SAM’s capabilities are limited by the complexity of the features in remote sensing images which includes irregular shapes and occlusions.

Experiments using text prompts present a powerful alternative tool for reducing the manual allocation of point prompts to each object in the image. However, the selection of words significantly impacted the object detection task and, consequently, the segmentation. A general text prompt was suitable for some sites since the model’s performance was highly context-dependent and the class characteristics varied from site to site. Therefore, an iteration of trial and error, using different text prompts and thresholds to optimize the segmentation performance was necessary. We evaluated a GPT-based prompt selection to explore other class descriptions we did not consider. However, results showed that, in most cases, this approach did not improve the results obtained with more general words chosen directly by the user.

Public datasets are often used to assess the performance of a model across different domains. The developers of the Samgeo package, Osco et al. [39], used drone and airborne imagery for its evaluation. For the segmentation task using point prompts, they report achieving a higher Dice coefficient (0.97) compared to text prompts (0.89) for the class “lake.” Our results expand the evaluation of the water class in rural environments, and consistently with the report, samples with clear boundaries also obtained a Dice coefficient performance above 0.9. Nevertheless, more challenging types are smaller water bodies present in these rural areas where most of our imagery was acquired and which are relevant for mosquito-vector control. When evaluating the performance of buildings, the Samgeo’s authors reported a higher Dice coefficient value (0.89) for the class “house” compared to “buildings” (0.70) using text prompts, which performed significantly better than the points. Similar to their findings, the use of “house” in our study had, in general, a better performance compared to the other alternatives. This may also be related that our focus was specifically on this type of building, and “buildings” implies a more general class. Another study by Ren [42], used the Inria and Deep Globe open datasets in urban areas, and tested bounding boxes and point prompts in building segmentation stating that SAM performance was variable, aligning with our results. A limitation of open datasets is the underrepresentation of low-middle income areas, as reported by Kucharczyk [43]. Initiatives such as Open Buildings aim to generate footprints from high-resolution satellite imagery to address this gap, highlighting the challenges of house detection in Africa using a CNN (U-Net) [44]. Our study evaluated samples from these low-income areas finding that characteristics of these buildings remain challenging for new pre-trained foundation models.

The use of the Samgeo package can provide several advantages to the end user. This includes the availability of a Graphic User Interface that can be executed on the Google Collaboratory cloud-based platform that helps overcome the limitations of computing resources and advanced programming skills. This alternative offers users an initial approach to assess the suitability of these tools for their study area. Regarding SAM, its generalization capability seems to underperform in some specific domains such as remote sensing [45] or medical imaging [46] due to the imagery characteristics. To enhance its domain-specific segmentation accuracy, pre-trained CNN models are being used to provide prompts [45], which increase the complexity for practical purposes or can result in variable performance compared to a supervised CNN model [42].

Previous work has followed different workflows on the use of drone in habitat mapping [31, 47, 48]. However, from an operational perspective, there is no clear framework for managing the image processing steps after generating the orthomosaic. SAM is an alternative in this workflow, however, the suitability of using this technology will depend on the characteristics of the vector ecology. It may be more appropriate for mosquito species that breed in discernible objects in drone imagery, as opposed to those that breed in leaf axils, tree holes and tyres [33]. Moreover, there are other constraints of the aerial imagery itself related to occlusion, e.g., water bodies under canopy trees, puddles covered by shadows, and water containers under roofs. Therefore, ground-based surveys are still required, and the use of drone technology can complement this methodology especially in difficult to access areas.

The application of drone technology for vector control purposes, ranging from habitat monitoring to larvicide application, is being considered in various countries and the use of a broadly trained model for automating the digitalization of the images can assist in these diverse tasks. While our study focused on malaria vectors, this can be expanded to other mosquito species such as Aedes, which are vectors for dengue that breed in water containers and the utility of using drones for breeding sites detection has been explored by Valdez-Delgado [49] and Passos [50]. The automatic identification of potential larval habitats could also guide drone larvicide spraying [51] which its applicable in the control of other vectors and can be extended to waterborne diseases in general. Furthermore, the ability to rapidly detect houses and informal settlements may be useful for broader risk mapping [52] and health planning activities. Overall, this technology can benefit interventions where an aerial view provide critical information. The availability and advances of automatic segmentation such as SAM can assist in the overall analysis, for example after natural disaster events such as earthquakes [53] or floods where a damage assessment is needed for a prompt response.

Additional research is needed to improve the performance of these models to ensure robustness for practical utilization. Future advances of accurate text guided object detectors, such as Grounding Dino, customized on high-resolution images for land classification will leverage the flexibility on locating the different habitats depending on the vector ecology. Moreover, considering images from underrepresented areas is crucial to improve the identification of rural human settlements. In this study, we use RGB images, which can be considered as a low-cost alternative compared to a multispectral image acquisition. However, although RGB images are easier and cheaper to collect, multispectral data may improve performance, particularly the Near-Infrared band, as it has shown an increased performance using CNN-based models [54]. Regardless of the differences between CNN and ViT models (such as SAM), adding multispectral images to future foundation models may increase the performance of water segmentation. Additionally, SAM’s capability relies on its extensive training on 11 million natural images [28], therefore, following work should be extended adding remote sensing imagery, since its performance has been reported to improve with additional data samples (one-shot learning) [39], and model adaptation to this domain [41].

From an operational point of view, further studies are needed to assess the ground-truth accuracy and cost-effectiveness of these approaches to have better understanding on how these methods can be included in disease control strategies [55]. The availability of software packages in GIS platforms can bring this type of technology closer to non-expert users and would help bridge the gap between new computer vision advancements and their application in real context. This study relied on retrospective data, therefore, additional research focused on the integration of these image analysis steps are needed to delineate a framework that can cover from the drone image collection to automatically mapping the targeted classes relevant for disease monitoring.

Although we covered five different countries, a limitation of this study is that we only sampled seventy patches of the landscape in total. Further analysis is required to assess the application in diverse contexts including the evaluation of other land classes (i.e. rice crops, drains) relevant for vector control purposes.

Despite this limitation, this study is the first to assess how foundation models can support analysis of high-resolution Earth observation data for vector control. With increasing levels of environmental change, monitoring these changes and quickly obtaining accurate information is essential for disease control programmes. These results illustrate the potential of foundation models to rapidly analyse imagery across different malaria-endemic settings and highlight the need for further assessments on their use for operational disease control.

## Conclusions

The purpose of this study was to provide insights on the segmentation performance of the Samgeo package in drone imagery surveillance for mosquito borne diseases. The presented results show that this technique is a potential tool to assist manual digitalization. However, it still requires the user to manually assess and set prompts and thresholds to achieve proper segmentation depending on the site’s own characteristics. Further evaluation and tests of these parameters are needed if this model is intended to be used, especially in aerial imagery from rural areas.

### Electronic supplementary material

Below is the link to the electronic supplementary material.

Supplementary Material 1

## Data Availability

The data that support the findings of this study are available upon reasonable request and approval from the relevant ethic committees to the corresponding authors. The code used is publicly available at https://github.com/fedra007/SAMgeo_malaria.

## Abbreviations

CNNs: Convolutional Neural Networks
UAV: Unoccupied Aircraft System
PPV: Positive Predictive Value
TPR: True Positive Value
RGB: Red, Green and Blue
