# Supplementary material for: Using image segmentation models to analyse high-resolution earth observation data: new tools to monitor disease risks in changing environments
Source: Int J Health Geogr. 2024 May 19;23:13. doi: 10.1186/s12942-024-00371-w (PMC11102859; doi:10.1186/s12942-024-00371-w)
Supplement: Supplementary file 1 — Supplementary Material 1 [file 12942_2024_371_MOESM1_ESM.pdf]

Supplementary information

1. Burkina Faso UAV samples (120x120m)

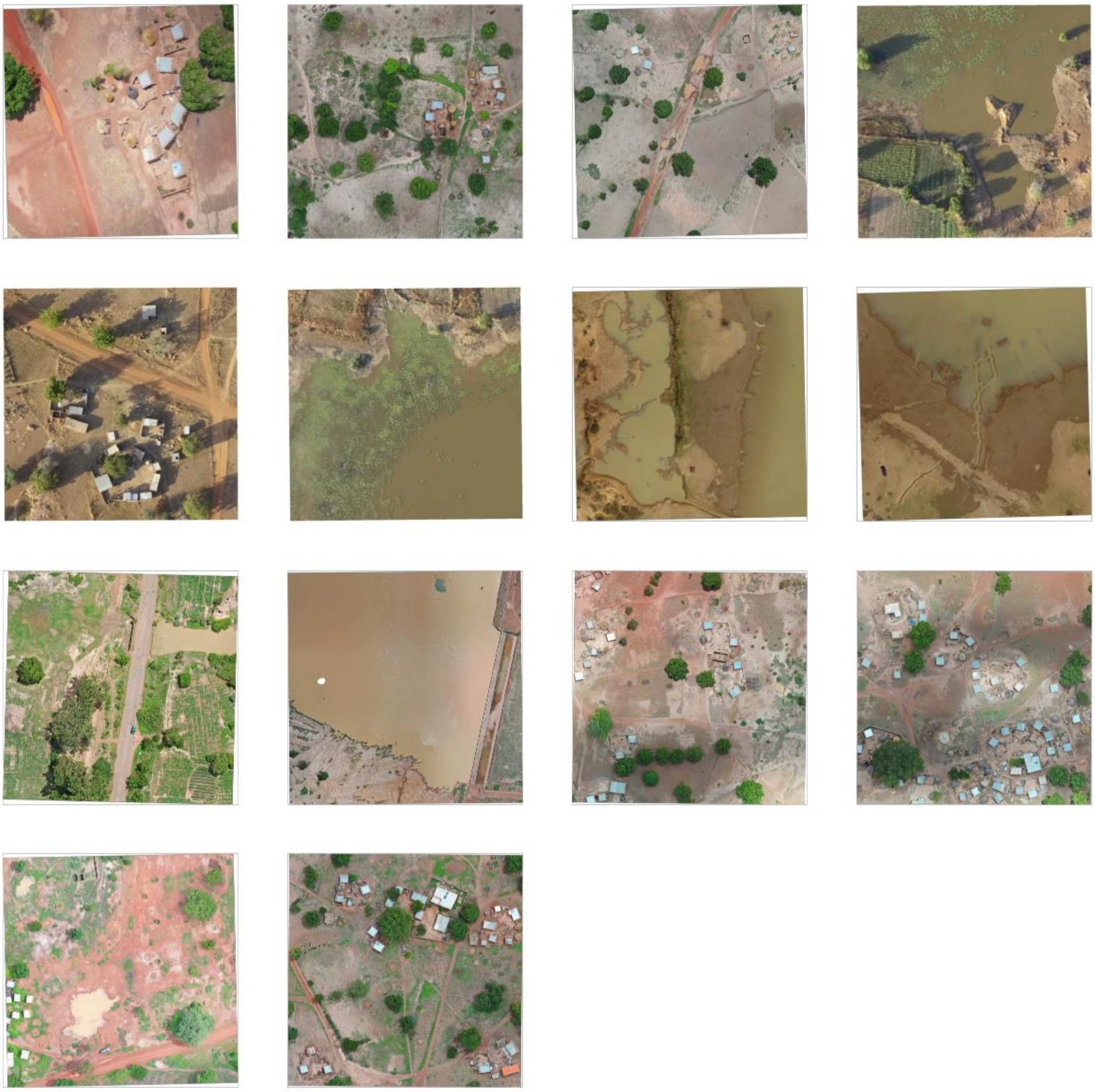

Point prompts:

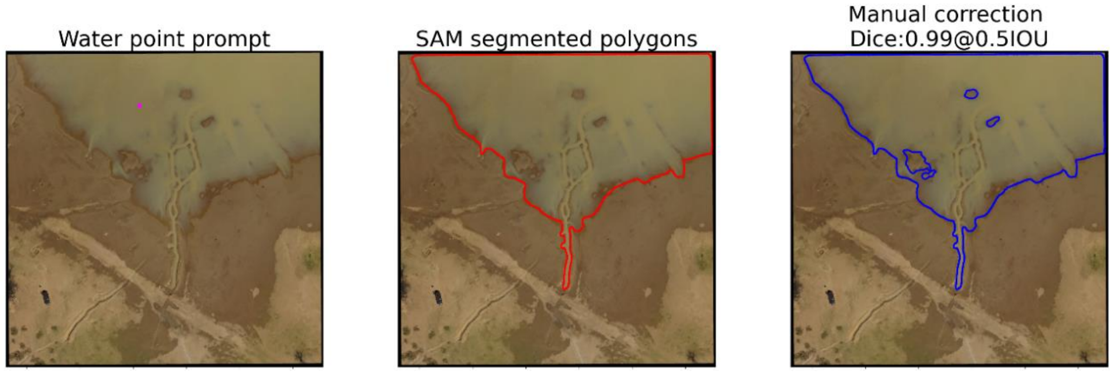

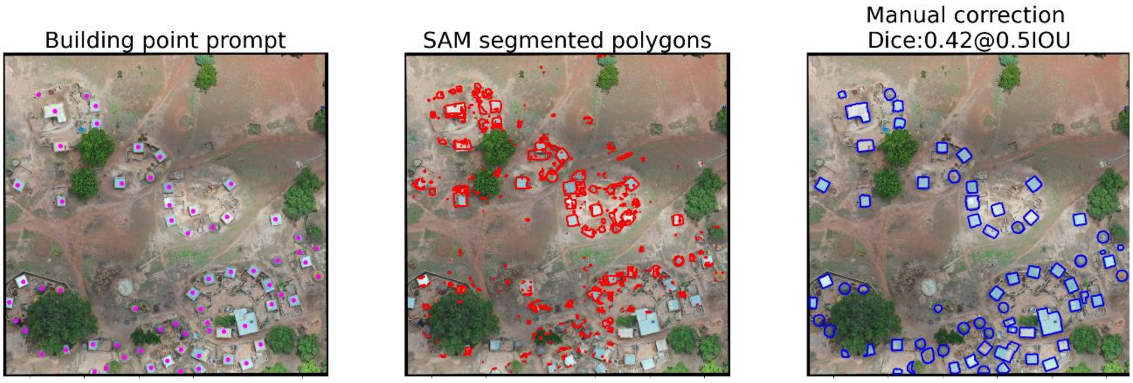

Text prompt: “Puddles”

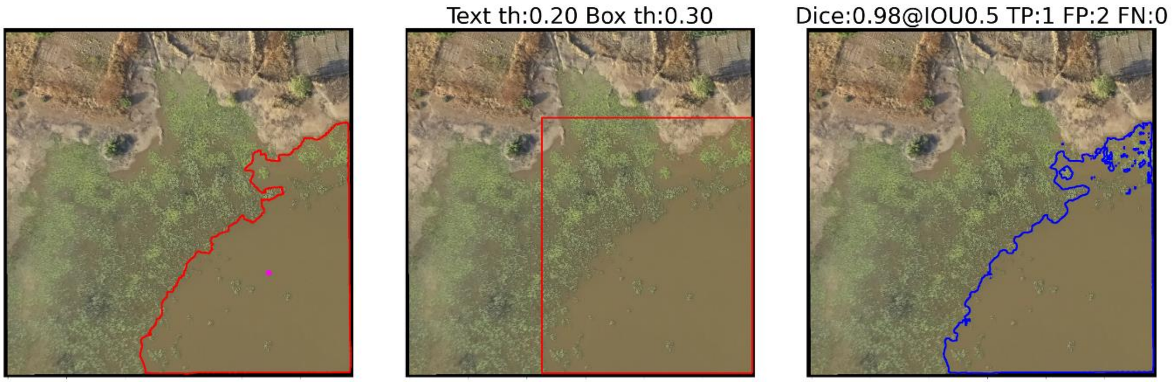

Text prompt: “House”

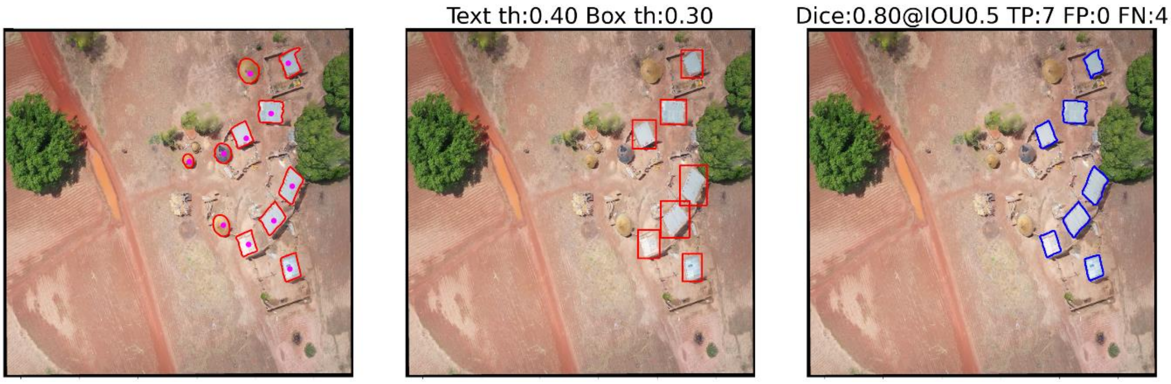

2. Ivory Coast UAV samples (120x120m)

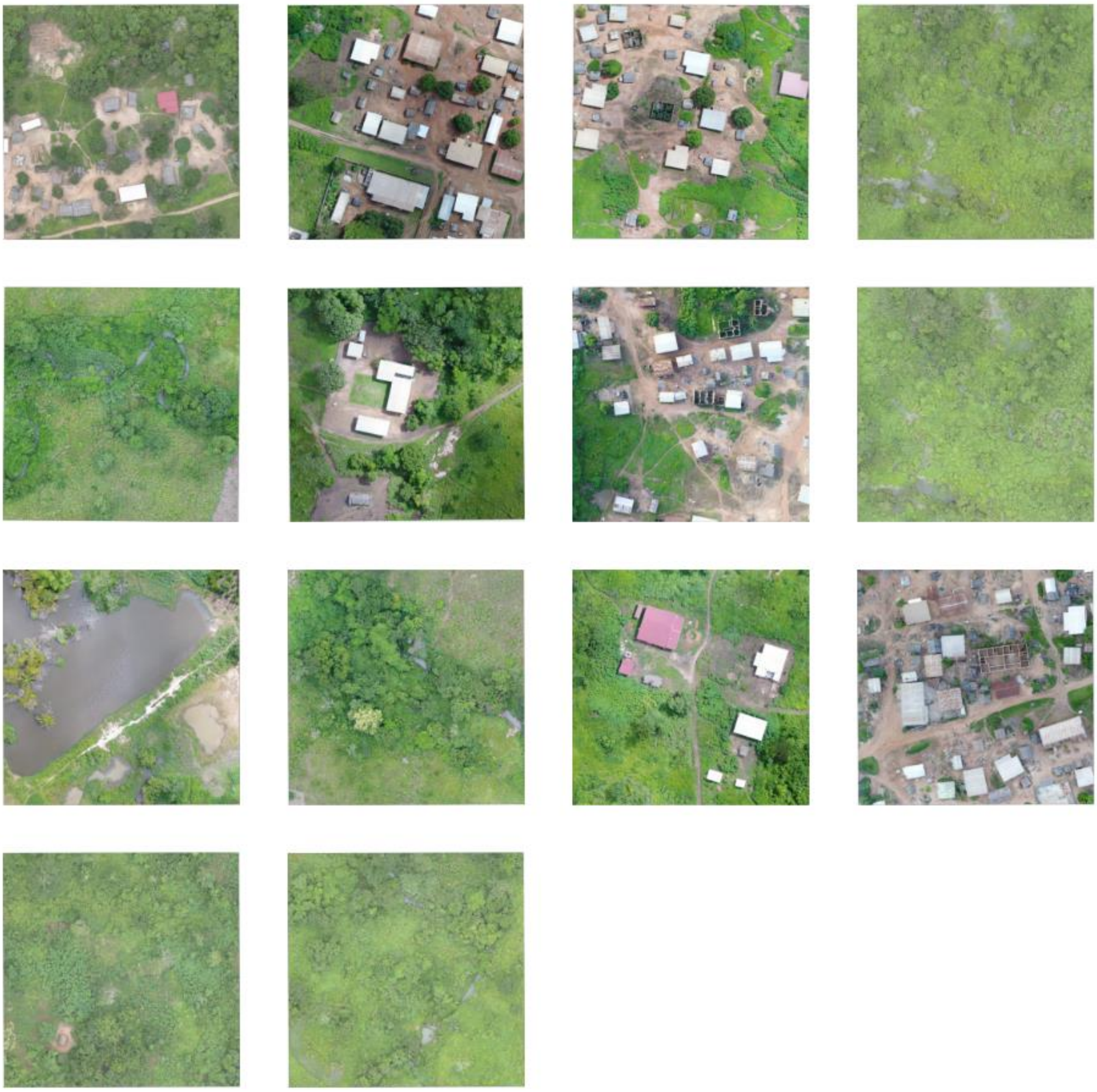

Ground photos of water bodies:

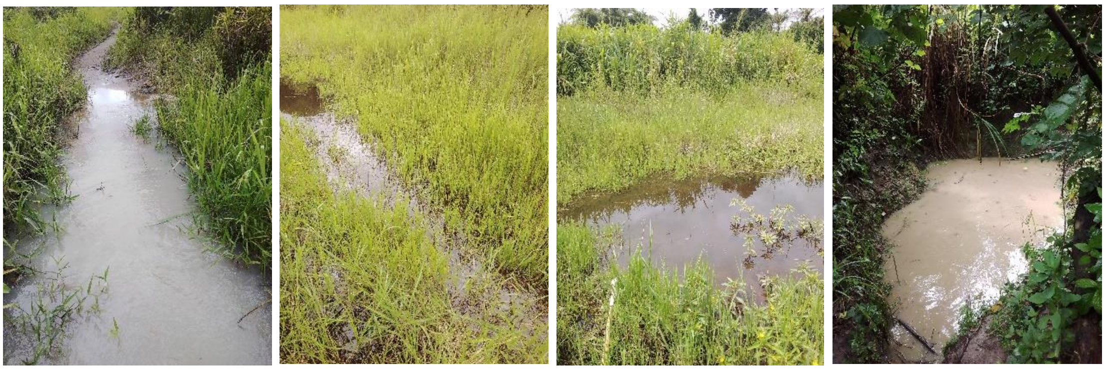

Point prompts:

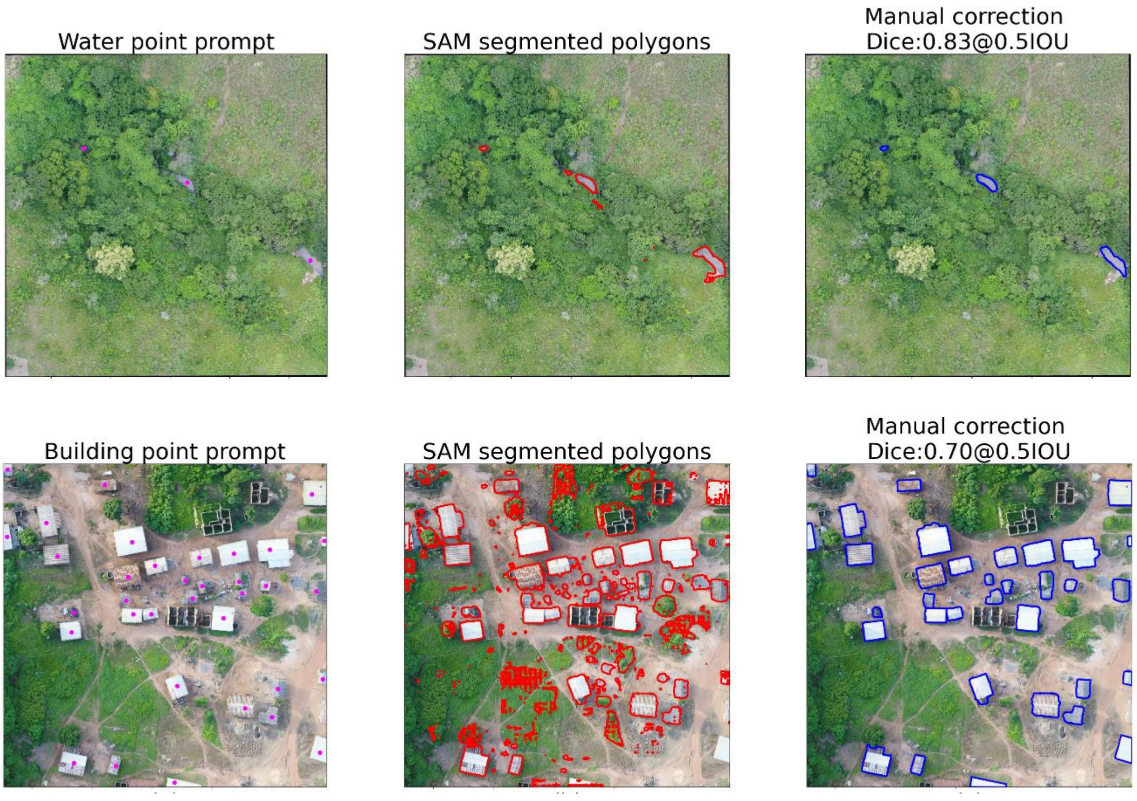

Text prompt: “Puddles”

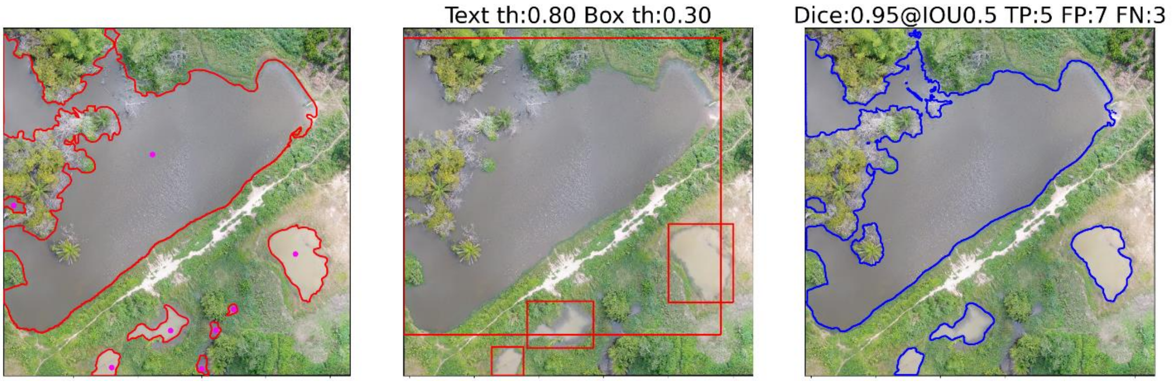

Text prompt: “House”

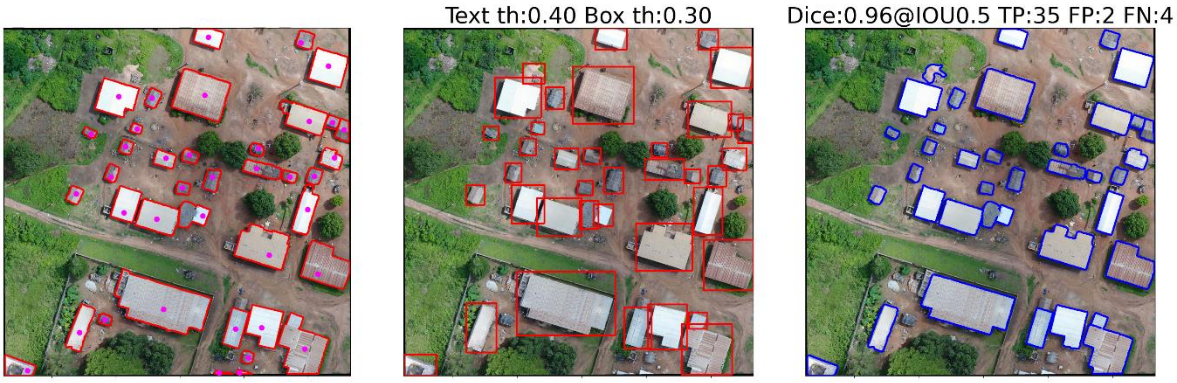

3. Peru UAV samples (120x120m)

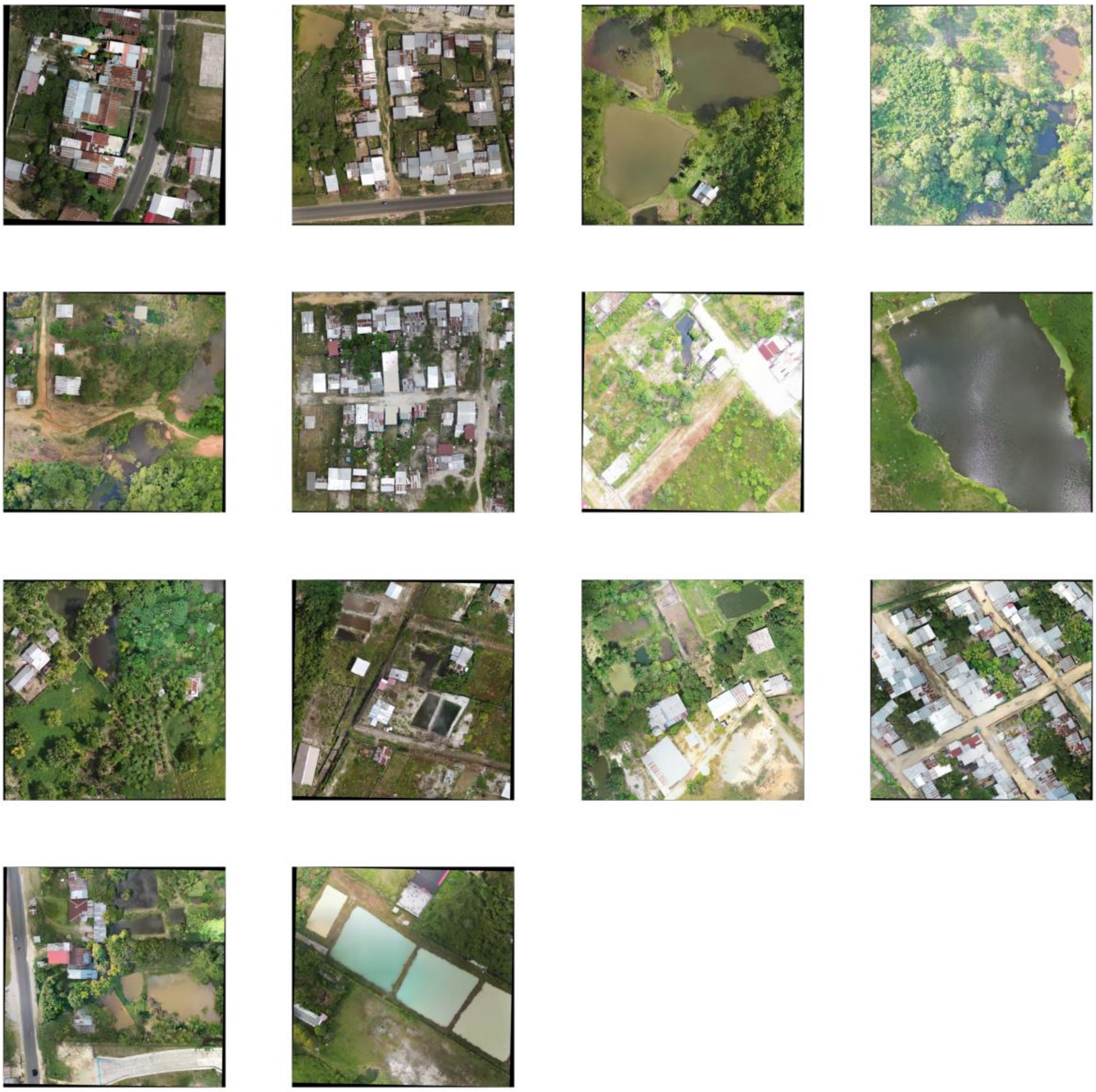

Ground photos of water bodies:

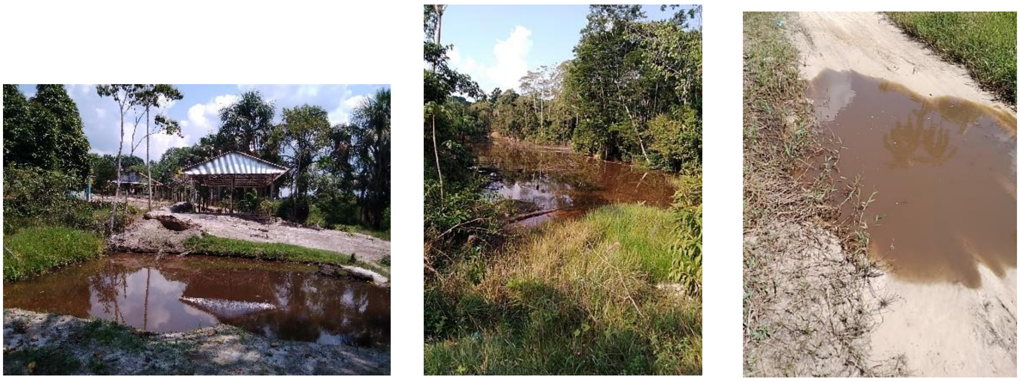

<u>Point prompts:</u>

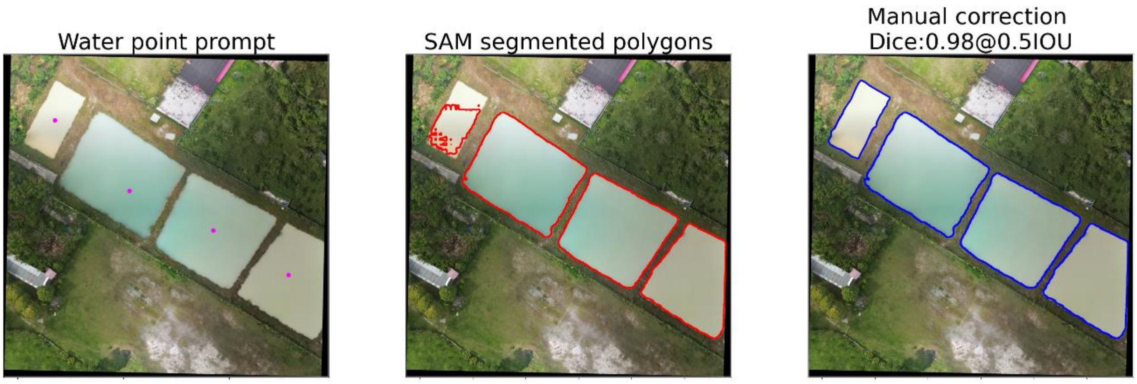

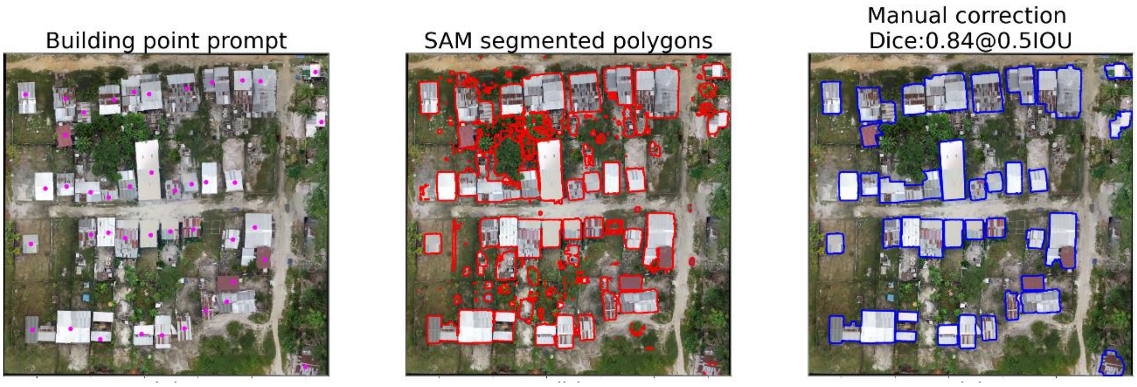

Text prompt: "Puddles"

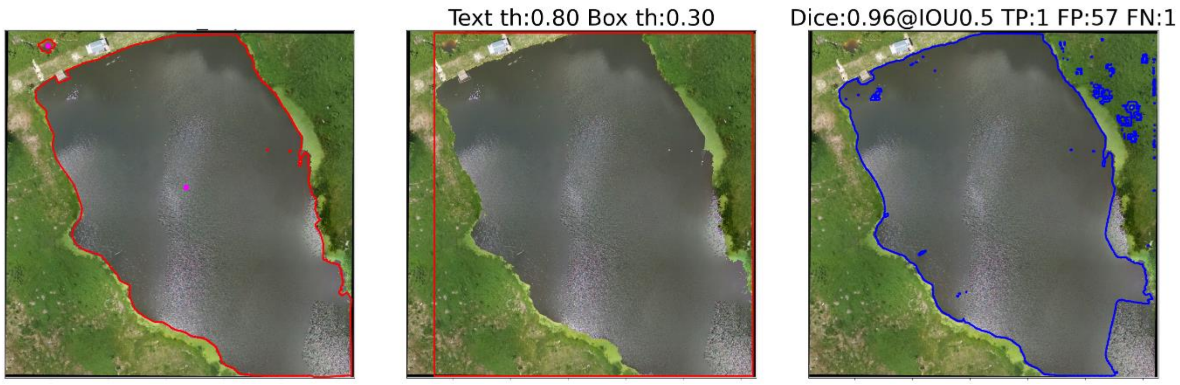

Text prompt: "House"

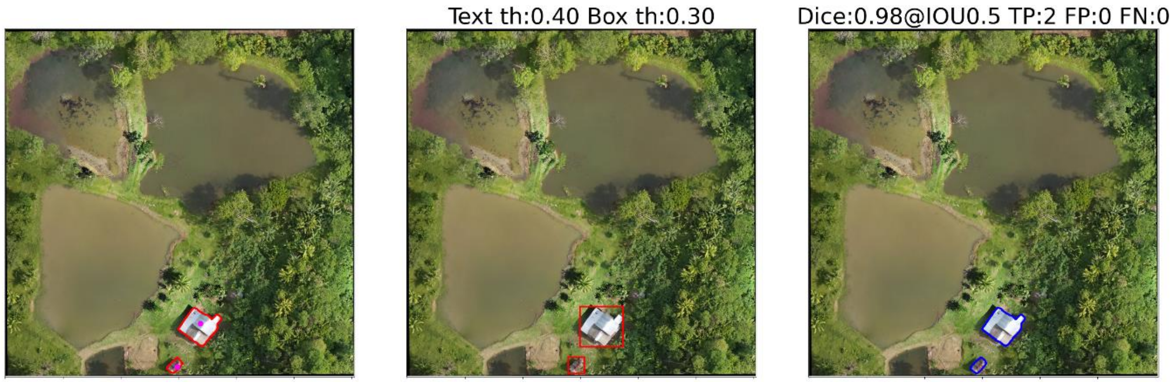

4. Malaysia UAV samples (120x120m)

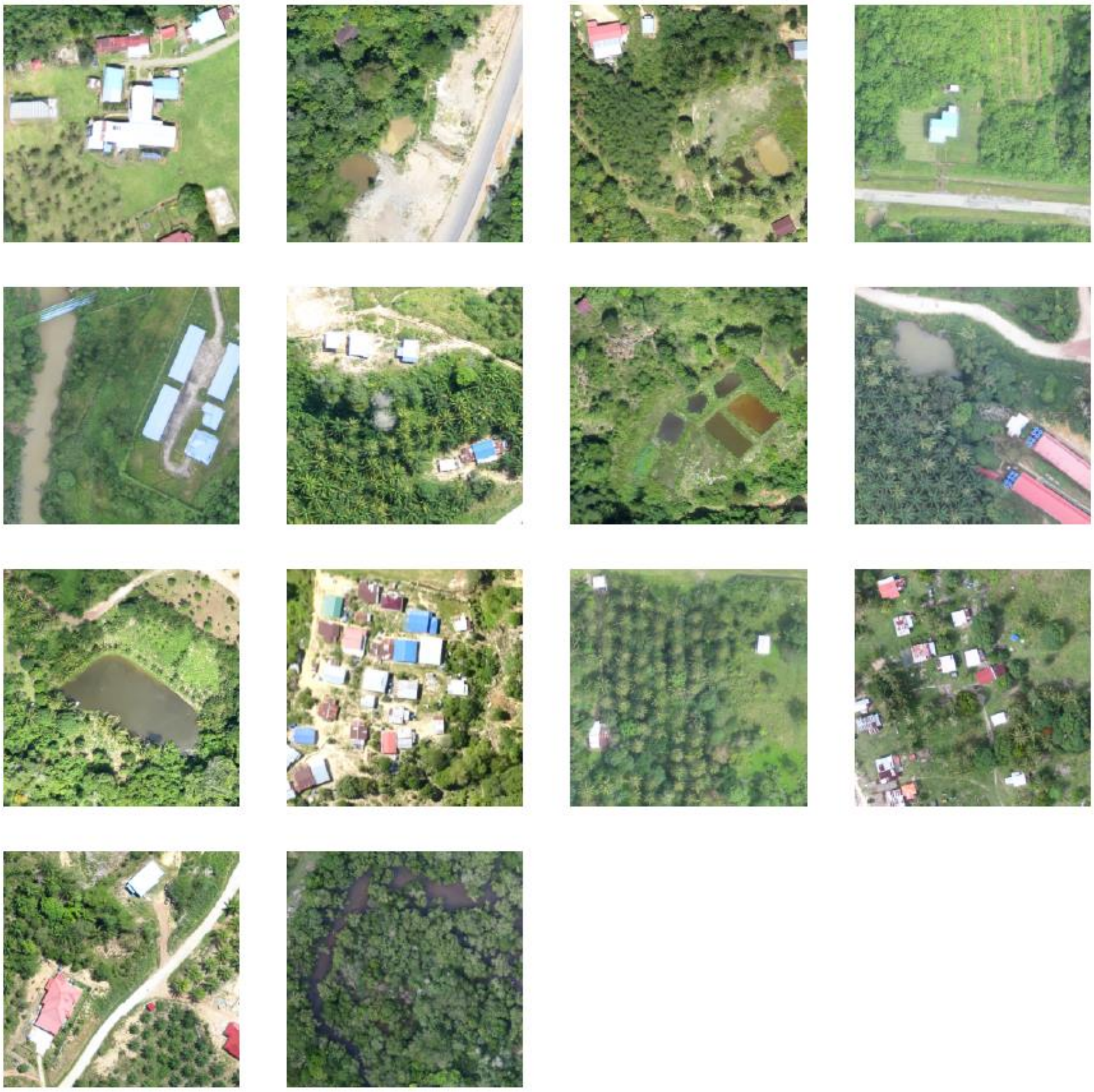

Point prompts:

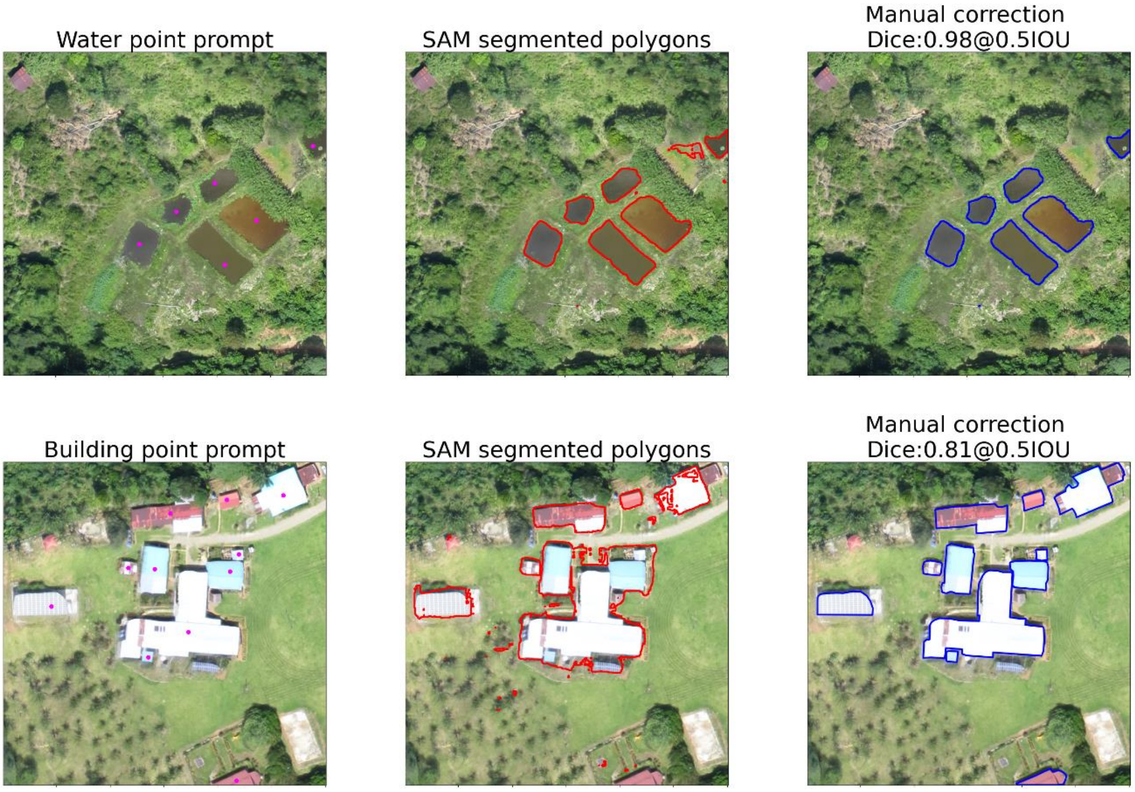

Text prompt: “Puddles”

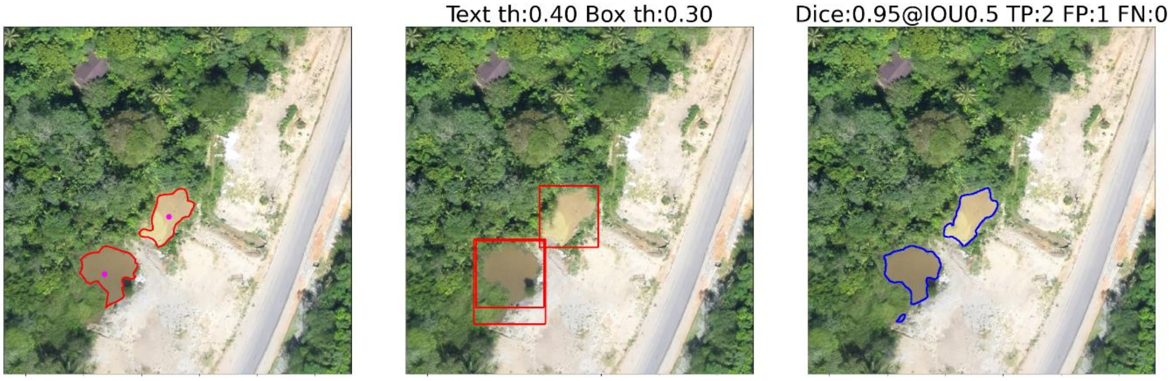

Text prompt: “House”

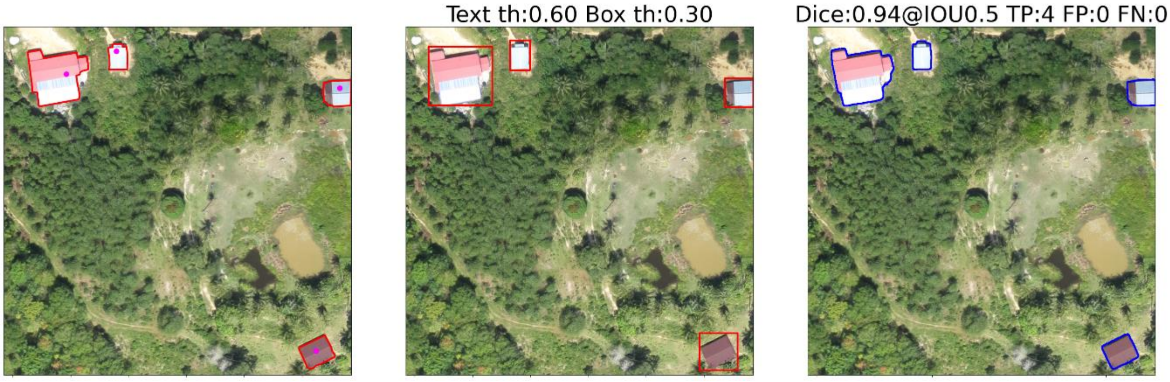

5. Tanzania UAV samples (60x60m)

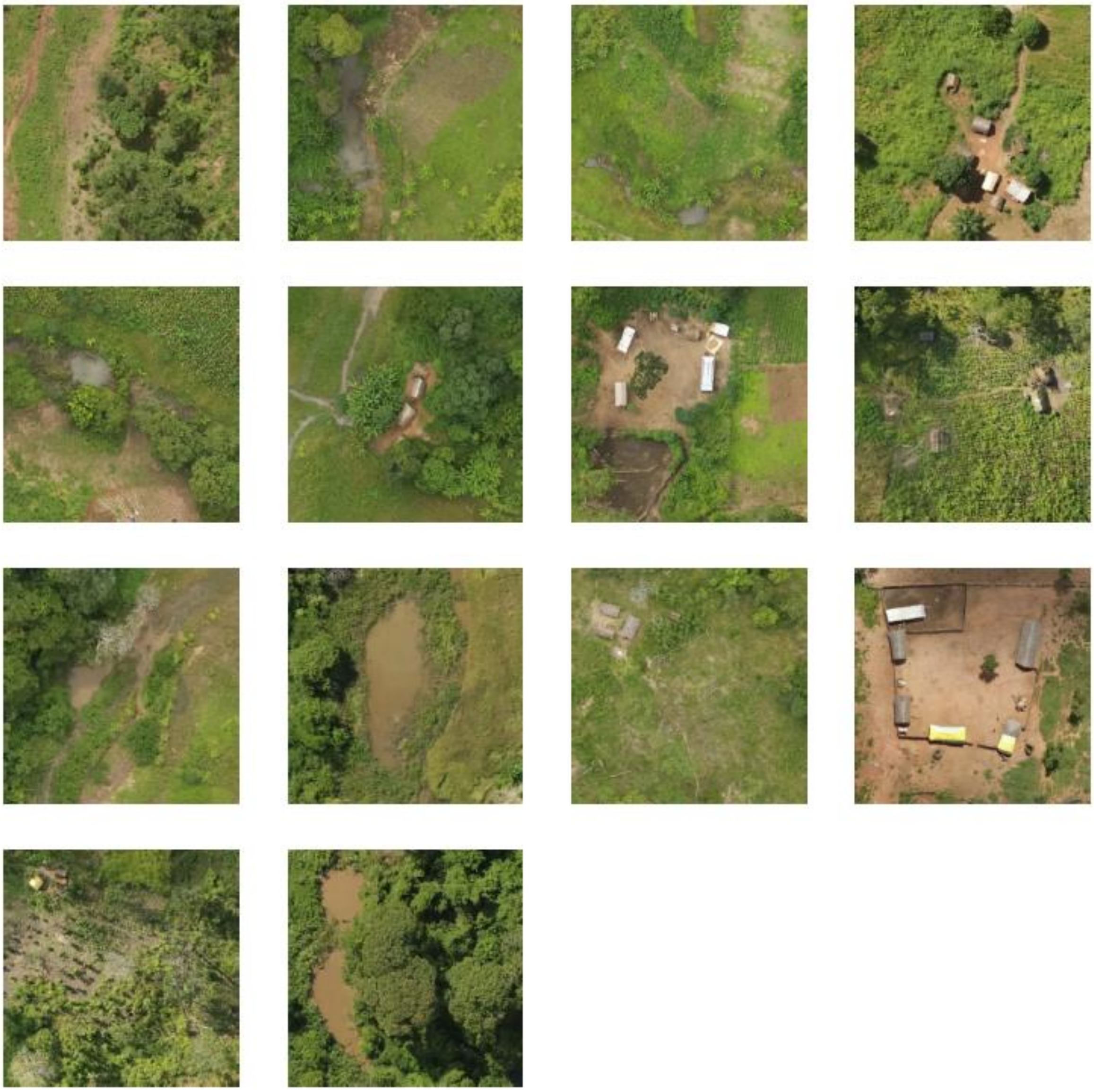

Ground photos of water bodies:

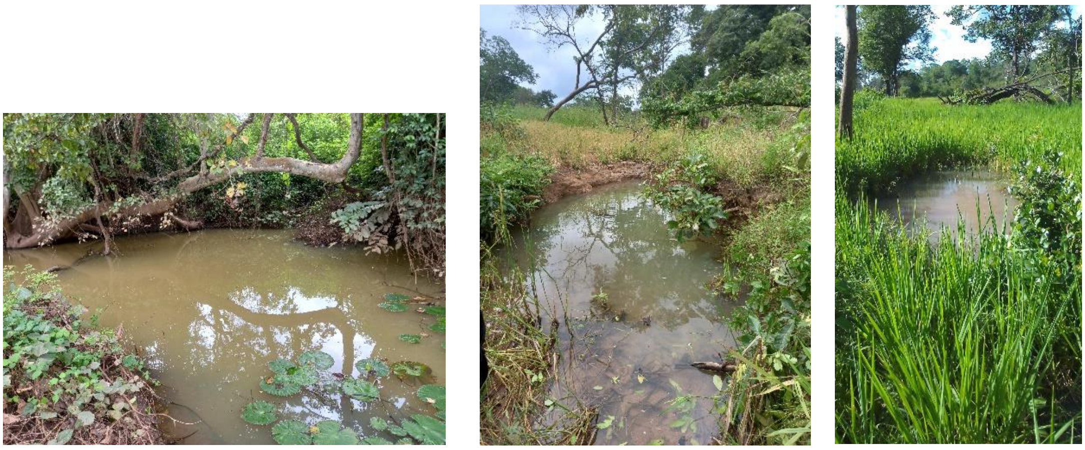

Point prompts:

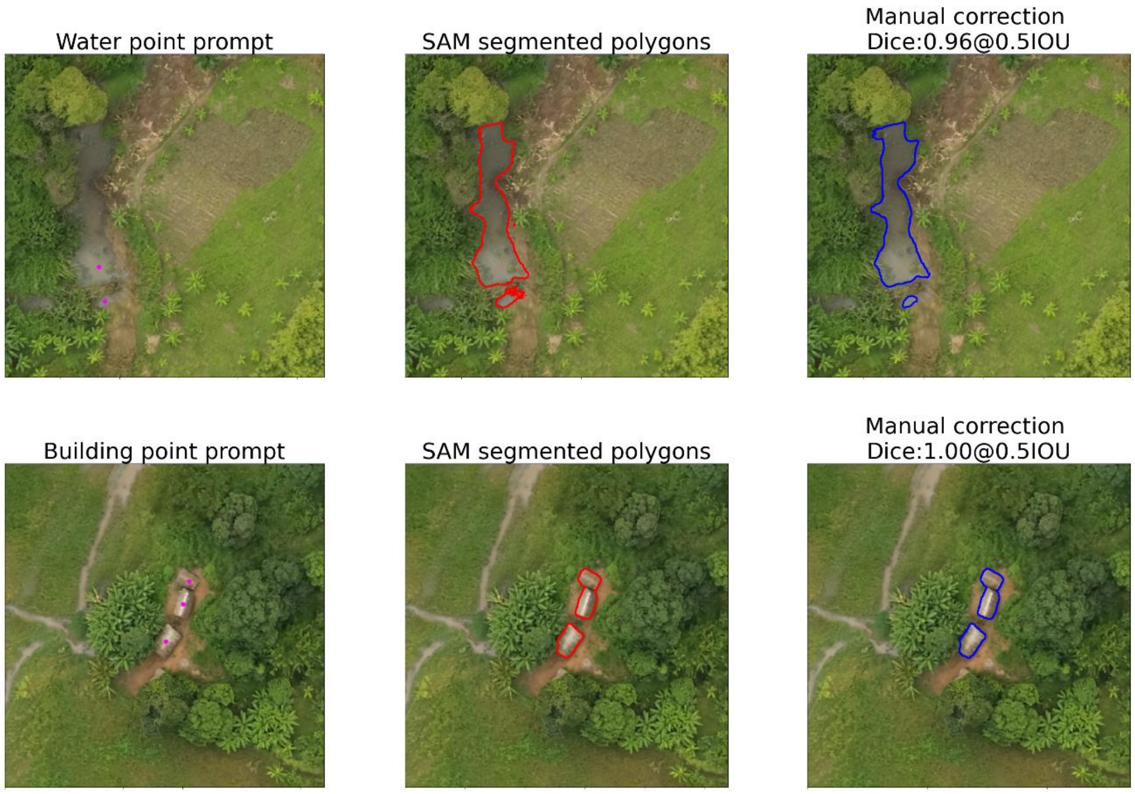

Text prompt: “Puddles”

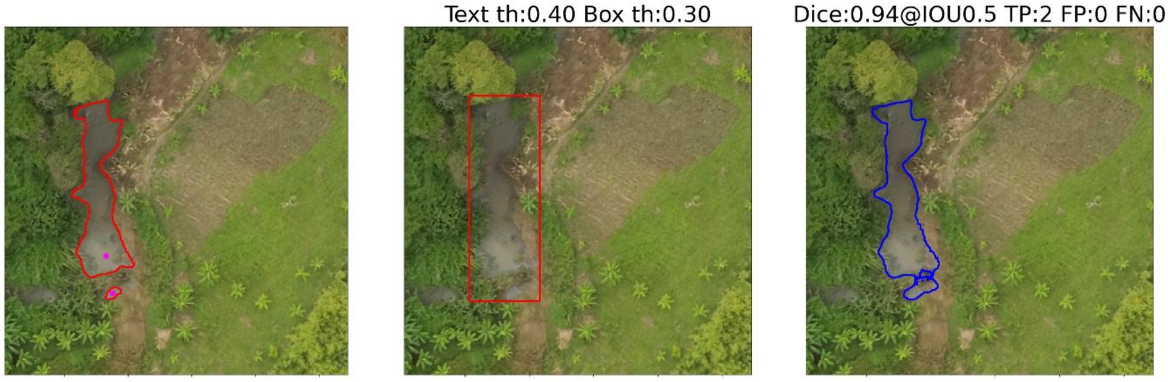

Text prompt: “House”

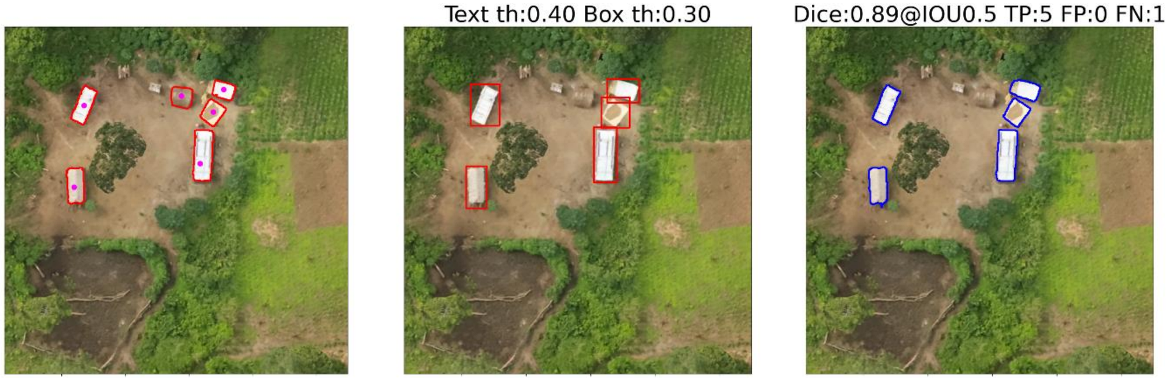

Chat GPT-4 Text queries and responses

Understanding the image (step 1 & 2)

Input image. Prompt: "Describe the uploaded image"

Output: "The image is an aerial view of a rural landscape that includes several ponds and a single building. The ponds are various sizes and shapes, with one larger body of water taking up a significant portion of the frame. The water appears murky, suggesting they might be man-made or for agricultural use. Surrounding the ponds are areas of greenery with a mixture of trees and open grassland. In the bottom right corner, there is a building with a white roof, which could be a small "House" or farm structure. There are no visible roads leading directly to the building, but there is a narrow path that connects the ponds and the building. The topography is mostly flat, and the entire scene is bathed in natural light, indicating a sunny day. The shadow cast by the trees on the water indicates that the sun is at a moderate angle, suggesting either morning or afternoon."

Input image. Prompt: "Describe the uploaded image"

Output: The image is an aerial view of a rural area with a collection of buildings. The terrain appears to be dry with reddish-brown earth and sparse green vegetation. There are several structures with different roofing materials, some with metallic roofs reflecting sunlight, and others with darker roofing that could be made of tiles or thatch. The layout seems unplanned, with buildings scattered in a non-uniform pattern. A dirt road runs through the scene, with no visible vehicles. The area is not densely populated, and the overall impression is of a small, rural village or community.

Input image. Prompt: "Describe the uploaded image"

Output: The image appears to be an aerial or satellite view of a tropical or subtropical area with dense vegetation. There are numerous trees with a distinctive crown shape, which could be palm trees, indicating that the area may be a plantation or a naturally palm-rich region. There are at least three structures visible: two with bright white roofs that stand out against the greenery, and one with a red roof, possibly indicating a residential or farm building. The layout doesn't seem to follow a strict pattern, suggesting that the area is not heavily developed or managed. The dense foliage and lack of visible roads or paths give the area a secluded feel.

Refining the prompt (step 3 & 4)

Input prompt: "Generate a 'text prompt' for the SAM model in order to segment water bodies in similar images to the one uploaded."

Output: "Segment the water bodies in an aerial rural landscape image. Identify and delineate all areas with visible water, including ponds and any other water features. The ponds are of various sizes and shapes, with murky water that could suggest agricultural use. The surrounding landscape includes a mix of trees and open grassland, with a single white-roofed building in the bottom right. Consider the variable lighting conditions and shadows cast by the trees on the water. Ensure to differentiate between water bodies and shadowed areas on the ground. Focus on the contrast between the greenery of the land and the brownish hue of the water to accurately segment the water bodies."

Input prompt: "Consider that the prompt for the model should be a list of keywords, each separated by a period. Generalize it to different types of water bodies."

Output: "Aerial view. Water segmentation. Ponds. Lakes. Rivers. Streams. Murky water. Clear water. Reflections. Water boundaries. Vegetation edges. Man-made water bodies. Natural water bodies. Shadow distinction. Water color variations. Land-water contrast."

Final list (manual selection): **"Ponds. Lakes. Rivers. Streams. Murky water. Clear water. Reflections."**

===

Input prompt: "Generate a 'text prompt' for the SAM model in order to segment buildings in similar images to the one uploaded."

Output: "Generate a segmentation map for an aerial image of a rural landscape. Identify and segment all the buildings which are characterized by their geometric rooftops in various materials, some with reflective metallic surfaces and others with darker matte surfaces. The buildings are distributed across a terrain with reddish-brown earth and pockets of sparse green vegetation. There is a visible dirt road bisecting the area. Exclude any non-building elements like the natural vegetation, the bare ground, and the road from the segmentation. Highlight each building with a distinct boundary and label them as 'Building' in the segmentation map. The goal is to create a clear distinction between man-made structures and the natural environment for analysis of rural development and land use."

Input prompt: "Consider that the prompt for the model should be a list of keywords, each separated by a period. Generalize it to different types of buildings."

Output: "Buildings. Roofs. Reflective surfaces. Matte surfaces. Geometric shapes. Metallic. Tiles. Thatch. Aerial view. Rural. Sparse vegetation. Reddish-brown earth. Dirt road. Segmentation boundaries."

Final list (manual selection): **"Buildings. Roofs. Reflective surfaces. Matte surfaces. Geometric shapes. Metallic. Tiles. Thatch."**

Table 3. Segmentation and object detection metrics according to text prompts

		0.2				0.5			
	Text prompt	Dice std.	CI	PPV	TPR	Dice std.	CI	PPV	TPR
Buildings	building	0.409	0.410 [0.409 0.411]	0.340	0.641	0.451	0.516 [0.515 0.517]	0.328	0.159
	house	0.425	0.494 [0.494 0.495]	0.424	0.656	0.415	0.585 [0.584 0.585]	0.418	0.211
	roof	0.378	0.282 [0.281 0.282]	0.268	0.570	0.457	0.402 [0.401 0.403]	0.171	0.037
	Ensembled	0.412	0.409 [0.408 0.410]	0.365	0.596	0.470	0.337 [0.336 0.338]	0.014	0.002
	GPT prompt	0.279	0.204 [0.203 0.204]	0.182	0.573	0.459	0.300 [0.299 0.301]	0.000	0.000
Water	puddles	0.437	0.453 [0.452 0.454]	0.246	0.421	0.494	0.427 [0.426 0.428]	0.043	0.050
	ponds	0.412	0.324 [0.324 0.325]	0.244	0.488	0.488	0.460 [0.460 0.461]	0.077	0.080
	water body	0.408	0.305 [0.304 0.305]	0.236	0.467	0.487	0.466 [0.466 0.467]	0.114	0.087
	Ensembled	0.443	0.570 [0.570 0.571]	0.270	0.442	0.490	0.422 [0.421 0.422]	0.046	0.036
	GPT prompt	0.436	0.608 [0.607 0.609]	0.249	0.390	0.484	0.372 [0.371 0.372]	0.000	0.000

Table 4. Segmentation and object detection metrics according to text prompts

		0.6				0.8			
	Text prompt	Dice std.	CI	PPV	TPR	Dice std.	CI	PPV	TPR
Buildings	building	0.467	0.393 [0.393 0.394]	0.121	0.033	0.471	0.329 [0.329 0.330]	0.000	0.000
	house	0.442	0.477 [0.476 0.478]	0.262	0.084	0.470	0.337 [0.336 0.338]	0.014	0.002
	roof	0.467	0.356 [0.355 0.357]	0.057	0.010	0.470	0.337 [0.337 0.338]	0.014	0.002
	Ensembled	0.471	0.329 [0.328 0.329]	0.000	0.000	0.471	0.329 [0.328 0.330]	0.000	0.000
	GPT prompt	0.471	0.329 [0.328 0.330]	0.000	0.000	0.471	0.328 [0.328 0.329]	0.000	0.000
Water	puddles	0.488	0.386 [0.385 0.387]	0.014	0.014	0.484	0.371 [0.371 0.372]	0.000	0.000
	ponds	0.494	0.426 [0.425 0.427]	0.032	0.039	0.490	0.399 [0.398 0.400]	0.014	0.021
	water body	0.495	0.453 [0.452 0.454]	0.061	0.044	0.490	0.399 [0.398 0.400]	0.014	0.021
	Ensembled	0.484	0.371 [0.371 0.372]	0.000	0.000	0.484	0.371 [0.370 0.372]	0.000	0.000
	GPT prompt	0.484	0.371 [0.371 0.372]	0.000	0.000	0.484	0.371 [0.370 0.372]	0.000	0.000
